# Smoking, drinking, and physical activity among Korean adults before and during the COVID-19 pandemic: a special report of the 2020 Korea National Health and Nutrition Examination Survey

**DOI:** 10.4178/epih.e2022043

**Published:** 2022-04-25

**Authors:** Sunhye Choi, Jinwook Bahk, Suyeon Park, Kyungwon Oh, Kyunghee Jung-Choi

**Affiliations:** 1 Division of Health and Nutrition Survey and Analysis, Bureau of Chronic Disease Prevention and Control, Korea Disease Control and Prevention Agency, Cheongju, Korea; 2 Department of Public Health, Keimyung University, Daegu, Korea; 3 Department of Environmental Medicine, Ewha Womans University College of Medicine, Seoul, Korea

**Keywords:** COVID-19, Smoking, Drinking, Exercise

## Abstract

**OBJECTIVES:**

This study aimed to describe trends in health behaviours between 2011 and 2020 and compare the changes in these behaviours between the 2020 COVID-19 pandemic and previous periods according to socio-demographic variables.

**METHODS:**

This study used data from the 2011 to 2020 Korea National Health and Nutrition Examination Survey. Current cigarette smoking, high-risk drinking, and inadequate physical activity levels were used as health behaviour indicators. The age-standardized prevalence, differences in prevalence between the periods, and the annual percentage change (APC) were calculated.

**RESULTS:**

Current cigarette smoking showed a decreasing trend (APC, -2.6), high-risk drinking remained unchanged, and inadequate physical activity levels increased (APC, 3.5) during 2011-2020. There were significant differences in high-risk drinking (3.1%p; 95% confidence interval [CI], 0.3 to 5.9) and inadequate physical activity levels (4.3%p; 95% CI, 0.4 to 8.1) between 2019 and 2020 in men. Among men, increased high-risk drinking was found in those aged 40-49 years, non-single households, urban residents, and the middle and highest income groups between 2019 and 2020. The low educational group and manual workers among men aged 30-59 years also showed an increased proportion of high-risk drinking. Inadequate physical activity levels also increased among men between 2019 and 2020 in those aged 30-39 years, non-single households, urban residents, and the upper-middle-income group.

**CONCLUSIONS:**

In the first year of the COVID-19 pandemic, Korean men’s high-risk drinking and inadequate physical activity levels increased. In addition to social efforts to reduce the spread of infectious diseases, active measures to positively change health behaviour are needed.

## GRAPHICAL ABSTRACT

[Fig f2-epih-44-e2022043]

## INTRODUCTION

Coronavirus disease 2019 (COVID-19) is an infectious disease caused by a novel zoonotic virus called severe acute respiratory syndrome coronavirus 2 (SARS-CoV-2). COVID-19 caused the third major coronavirus outbreak in the 21st century, after severe acute respiratory syndrome and Middle East respiratory syndrome [1]. Since the first outbreak in Wuhan, China, in December 2019, the disease has rapidly spread worldwide, infecting 380,321,615 people and causing 5,680,741 deaths as of February 2, 2022 [2]. The World Health Organisation (WHO) declared an international public health emergency on January 30, 2020, and raised the global risk to “very high” on February 29, 2020, before declaring a pandemic on March 11, 2020 [3]. While COVID-19 vaccines and treatments have been developed, new mutations of SARS-CoV-2 have emerged, and the pandemic continues. In Korea, the first case of COVID-19 occurred in January 2020, and 907,214 people have been infected and 6,812 have died as of February 3, 2022 [4].

The Korean government has implemented various non-pharmaceutical interventions, such as social distancing, diagnostic testing, contact tracing, isolation, and quarantine after the beginning of the 2020 pandemic, as well as vaccinations starting in February 2021 [5,6]. In addition, “Tips for a Healthy Life During COVID-19” provides information for daily activities that can help people maintain a healthy physical, mental, and social life during the prolonged COVID-19 outbreak [7]. The WHO also recommended regular physical activity at home in the “Be Active” campaign and has suggested balanced nutrition for a healthy diet [8]. Smokers were advised to quit smoking because they often raise their hands to their mouths, which increases the risk of infection, and they are at risk of becoming seriously ill due to impaired lung function when infected with COVID-19 [8].

The Korea Disease Control and Prevention Agency (KDCA) reported some changes in health behaviours and overall health during the 2020 COVID-19 pandemic [9,10]. According to the 2020 Korea National Health and Nutrition Examination Survey (KNHANES), the current smoking rate and monthly drinking rate of adults have improved slightly compared to 2019, but physical activity has deteriorated [9]. The results of the 2020 Korea Community Health Survey also indicate that consumption of instant foods, soda, and delivery food increased, and physical activity, smoking, and drinking behaviour decreased compared to before the COVID-19 epidemic [10]. The smoking rate, drinking rate, and physical activity levels of adolescents decreased in the 2020 Korea Youth Risk Behaviour Survey, showing the same results as adults [11].

This study aimed to describe trends in the prevalence of current cigarette smoking, high-risk drinking, and inadequate physical activity levels between 2011 and 2020 and compare the changes in health behaviour in the 2020 COVID-19 pandemic versus the pre-pandemic periods according to demographic and socioeconomic variables.

## MATERIALS AND METHODS

### Data

This study used data from the 2011 to 2020 KNHANES, conducted by the Korea Centres for Disease Control and Prevention (KCDC), the predecessor of the KDCA. The KNHANES is a nationwide and representative cross-sectional survey that produces national statistics on the health and nutritional status of Koreans. The survey sample is extracted by applying 2-step stratified and clustered sampling using survey districts and households as the primary and secondary extraction units. The survey subjects are all household members aged 1 or older, from 20-25 households per survey district, with 192 survey districts every year. The annual survey participation rate is about 73% [12].

The KNHANES consists of health examinations, a health interview, and a nutrition survey. The health interview includes a faceto-face interview administered by trained interviewers (chronic diseases, healthcare utilisation, injuries, etc.) and a self-administered survey (smoking, drinking, etc.). The survey items differ for children (1-11 years old), adolescents (12-18 years old), and adults (19 years or older) [12]. Our analysis was limited to adults who participated in the 2011-2020 KNHANES. The numbers of study subjects from 2011 to 2020 are presented in Supplementary Materials 1-9.

### Variables

Current cigarette smoking, high-risk drinking, and inadequate physical activity levels were used as health behaviour indicators. The current cigarette smoking rate was defined as the proportion of people who had smoked 5 packs or more of cigarettes over their lifetime and currently smoked cigarettes. We used the question “Do you currently smoke?” from 2011 to 2018, which was changed to “Do you currently smoke cigarettes?” from 2019 to 2020. In 2005, the Alcohol Use Disorders Identification Test was introduced to the KNHANES to investigate the frequency of drinking, the amount of drinking, and the frequency of binge drinking. High-risk drinking was defined as drinking at least twice a week, with an average consumption of 7 drinks or more for men and 5 drinks or more for women. For physical activity, the Global Physical Activity Questionnaire (GPAQ) was introduced in 2014 to measure the level of physical activity in 3 domains (work, transport, and leisure time). The GPAQ consists of 16 questions, including time for moderate-intensity activity, vigorous-intensity activity, and sitting by each domain. Inadequate physical activity was defined as not practicing moderate-intensity and vigorous-intensity physical activity for a total of 150 minutes or more during a usual week, as calculated by adding up all active hours during work, transport, and leisure time.

Based on the number of household members, the subjects were divided into single-person households and households with 2 or more members. The residential area was grouped into urban and rural areas. We classified equivalised household income into quintiles, adjusted by the square root of household size. Considering the differences in education level by age, subjects aged 30-59 years were classified into high school graduates or less and college graduates or higher, and subjects aged 60 or older were classified into middle school graduates or less and high school graduates or higher. Occupation was categorised into 3 groups for those aged 30-59: non-manual (professional/clerical), manual (service/sales workers, skilled agricultural and fishery workers, crafts and related trades workers, plant and machine operators, or elementary occupations), and others (unemployed, students, military personnel, or homemakers).

### Statistical analysis

The prevalence of current cigarette smoking, high-risk drinking, and inadequate physical activity levels were age-standardized using SAS (PROC SURVEYREG). To adjust for differences in changes in the age structure of each year, the age-standardized prevalence was calculated using age-specific and gender-specific structures of the estimated population based on the 2005 Population Projections for Korea. Sampling weights assigned to participants were applied to all analyses to represent the Korean population. Sampling weights were generated considering the complex sample design, the non-response rate of the target population, and post-stratification. The prevalence according to age groups were crude rates calculated using SAS (PROC SURVEYMEANS). The prevalence according to socio-demographic variables other than age groups were age-standardized using SAS (PROC SURVEYREG).

The annual percentage changes (APCs) were calculated to identify trends from 2011 to 2020 for current smoking and high-risk drinking and from 2014 to 2020 for inadequate physical activity using the Joinpoint Regression Program. The differences between periods were calculated between 2011-2019 and 2020, between 2017-2019 and 2020, and between 2019 and 2020, respectively. The prevalence rates for 2011-2019 and 2017-2019 were obtained by combining the denominator and numerator of the data for each year. The difference in estimates between the periods was calculated using SAS (PROC SURVEYREG). All analyses were performed using SAS version 9.4 (SAS Institute Inc., Cary, NC, USA) and the Joinpoint Regression Program version 4.1.1.1 (US National Cancer Institute, Bethesda, MD, USA).

### Ethics statement

This study used only the de-identified database disclosed to the public without personal identification information; therefore, neither approval by the institutional review board nor obtaining informed consent was necessary.

## RESULTS

**Figure 1.**
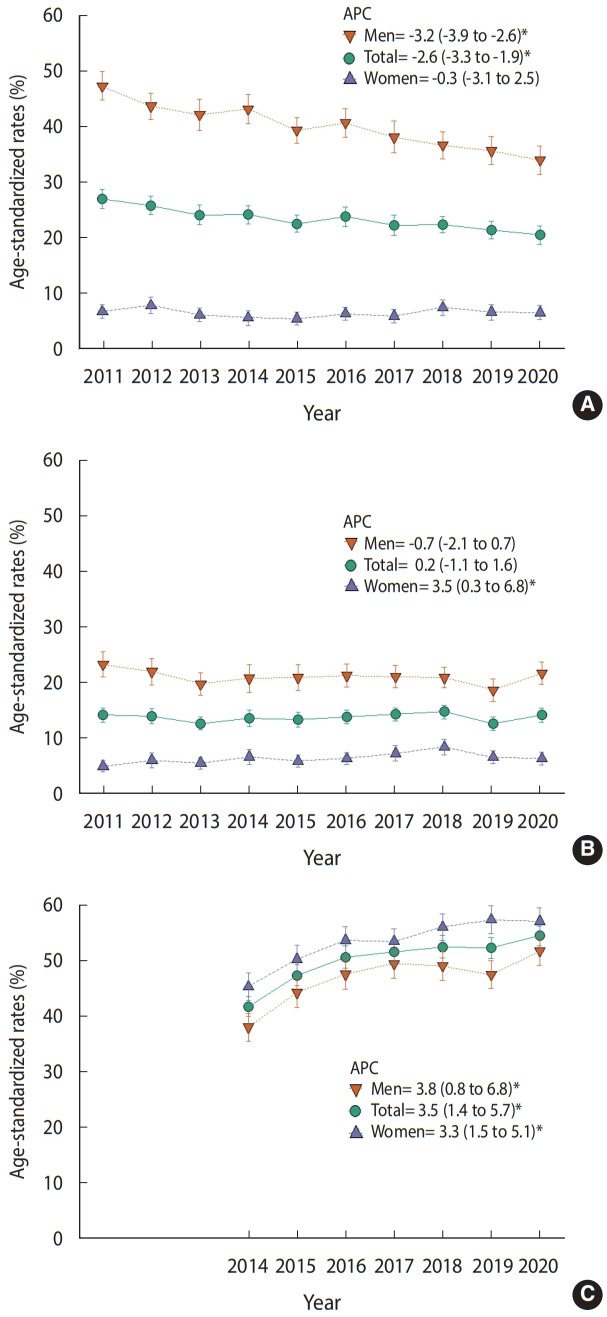
Trends of current cigarette smoking (A), high-risk drinking (B), and inadequate physical activity levels (C) by gender among Koreans aged 19 or older in the 2011-2020 Korea National Health and Nutrition Examination Survey. Values are presented as APC (95% confidence interval). APC, annual percent change. ^*^p<0.05.

Figure 1 shows the current cigarette smoking, high-risk drinking, and inadequate physical activity level trends from 2011 to 2020. In the total population, current cigarette smoking showed a decreasing trend (APC, -2.6; 95% confidence interval [CI], -3.3 to -1.9), high-risk drinking remained unchanged, and inadequate physical activity levels showed an increasing trend (APC, 3.5; 95% CI, 1.4 to 5.7).

The decline in the prevalence of current cigarette smoking was driven by men, with the men smoking rate in 2020 being 34.0% (Supplementary Material 2). There was no significant change in cigarette smoking rates for men (-1.8%p; 95% CI, -5.3 to 1.8) or women (0.0%; 95% CI, -2.0 to 2.0) between 2019 and 2020 (Table 1, Supplementary Material 10). For men, cigarette smoking rates according to most demographic and socioeconomic variables showed a decreasing trend from 2011 to 2020. However, there were no significant changes in smoking rates between 2019 and 2020 except for the lowest income group.

Table 2 and Supplementary Material 11 show the trends and comparisons between periods for high-risk drinking according to socio-demographic variables. In men, there was no significant change in the high-risk drinking rate from 2011 to 2020 (APC, -0.7; 95% CI, -2.1 to 0.7), but it increased significantly in 2020 compared to 2019 (3.1%p; 95% CI, 0.3 to 5.9). The men subgroups with significant differences in high-risk drinking between 2019 and 2020 were those aged 40-49 years old (6.9%p; 95% CI, 0.4 to 13.4), members of households with 2 or more members (3.9%p; 95% CI, 0.8 to 6.9), residents of urban areas (3.4%p; 95% CI, 0.3 to 6.5), those in the middle (6.9%p; 95% CI, 0.9 to 12.9) or highest (7.8%p; 95% CI, 1.4 to 14.2) income groups, high school graduates or less among those 30-59 years old (9.4%p; 95% CI, 2.8 to 16.0), and manual workers (6.6%p; 95% CI, 0.0 to 13.1). In women, high-risk drinking increased after 2011 (APC, 3.5; 95% CI, 0.3 to 6.8), but there was no significant difference between 2019 and 2020 (-0.3%p; 95% CI, -1.9 to 1.4).

From 2014 to 2020, inadequate physical activity levels increased significantly in both women (APC, 3.3; 95% CI, 1.5 to 5.1) and men (APC, 3.8; 95% CI, 0.8 to 6.8) (Table 3, Supplementary Material 12). In 2020, the rate of inadequate physical activity was 57.0% for women and 51.7% for men (Supplementary Materials 8 and 9). The inadequate physical activity rate increased significantly among men from 2019 to 2020 (4.3%p; 95% CI, 0.4 to 8.1), but not among women. There were significant differences in the inadequate physical activity rate between 2019 and 2020 for men aged 30-39 years (9.5%p; 95% CI, 1.2 to 17.9), those belonging to a household with 2 or more members (4.5%p; 95% CI, 0.4 to 8.7), residents of an urban area (4.4%p; 95% CI, 0.3 to 8.5), and those in the upper middle-income group (9.9%p; 95% CI, 2.3 to 17.6).

## DISCUSSION

Korea’s response to COVID-19 has been successful without a national lockdown [5]. There were 3 infection crises in February, August, and December of 2020, which were overcome with various timely interventions [13]. The testing-tracing-treatment strategy for a quick response was important [14], but citizens’ cooperation in social distancing was essential [15]. Although Korea achieved fewer confirmed cases and deaths and better economic performance than comparable countries, it could not completely escape the shadow of the pandemic. In 2020, the gross domestic product growth rate decreased by 3.7%p and the private consumption growth rate by 7.4%p, which was a shock equivalent to the 2008 global financial crisis [16]. Among various industries, face-to-face service industries such as arts, sports, and recreation services and the accommodation and food service sectors were hit particularly hard. About 460,000 Korean people lost their jobs in 2020 [16]. There were many unemployment struggles, especially among temporary or daily workers in the manufacturing industry and self-employed individuals with employees. We found that there were significant differences in high-risk drinking and inadequate physical activity levels between 2019 and 2020 in men.

COVID-19 may reduce alcohol consumption by restricting social interaction via social distancing policies [17] or increase alcohol consumption due to the stress and anxiety caused by the COVID-19 pandemic [18]. Although some studies in France and Canada reported decreased risky drinking behaviours [17,19], most studies reported increased alcohol intake [18,20-27], similar to this study. However, high-risk drinking increased only in men in Korea. The high-risk drinking rate of the overall population and women did not increase, and the proportion of subjects who responded that their alcohol consumption had decreased during the COVID-19 pandemic was higher than that of those who said it had increased [10]. The quantity of delivered liquor decreased slightly in 2020 [28]. We therefore assume that high-risk drinking habits were concentrated in some specific groups, such as middle-aged men with an educational level of high school or less and manual workers, who may have been hit harder by the COVID-19 pandemic than other groups [16]. The economic and psychological impact of the COVID-19 pandemic was concentrated in these groups, and they may have chosen to drink alcohol to reduce their increased stress and anxiety.

Although there are variations in study results depending on the country and study period, physical activity levels generally decreased during the COVID-19 pandemic [20,21,24,26]. A similar trend was found in Korea. No sweeping national lockdown was instituted in Korea in 2020, but social distancing was flexibly implemented according to the COVID-19 epidemic stage [5]. Depending on the social distancing level, the operation of sports facilities was suspended or restricted. The fact that the number of confirmed cases surged due to droplet exposure while exercising at indoor sports facilities in February 2020 received attention in the early stages of the COVID-19 pandemic, which led to a recognition of the dangers of indoor sports facilities during the pandemic [29]. In addition, the Korean government placed an emphasis on social distancing and asked people to avoid going out except for purchasing essential daily necessities, visiting medical institutions, and commuting [5]. All of these factors could reduce physical activity levels in Korea. In this study, the decrease in physical activity was significant only in men. Men typically engage in physical activity primarily through leisure time and work, whereas women may engage in physical activity through housework [30]. Thus, the impact of the COVID-19 pandemic seems to have had a greater impact on men’s physical activity levels.

The current cigarette smoking rate has decreased since 2011, and it decreased in 2020 compared to 2019, but this change was not statistically significant. A similar finding was reported in a previous study conducted in England [25]. Until now, most studies on health behaviour related to COVID-19 have been cross-sectional surveys that ask whether the amount of smoking increased or decreased during the COVID-19 pandemic. Some studies reported that the amount of smoking increased [19,25], but the proportions of the groups with an increased smoking amount and those with a decreased smoking amount were similar [25,26,31,32]. This suggests that the COVID-19 pandemic has increased the smoking amount in some individuals due to stress, anxiety, boredom, and other factors, and decreased it in those who quit to reduce their risk of COVID-19 [20,24,25,33]. The change in the amount of smoking in Korea should be further analysed using nationally representative repeated surveys, such as the KNHANES.

The questionnaire on e-cigarettes (ECs) and heated tobacco products (HTPs) changed significantly in 2019; therefore, this study did not address the use of those products. However, the consumption of ECs and HTPs in Korea has risen in recent years [33]. In the United States, a sharp increase in EC use has recently been reported in young people, and EC use was linked to subsequent cigarette smoking [34]. Therefore, further studies related to ECs and HTPs and the COVID-19 pandemic are warranted.

These study results should be interpreted carefully due to the study’s limitations. The trends and differences by period were analysed according to socio-demographic variables, but the CIs were wide due to the presence of relatively few subjects for each relevant variable.

## CONCLUSION

Despite some limitations, this study identified changes in health behaviours during the 2020 COVID-19 pandemic while taking into account long-term trends using nationally representative data. Despite Korea’s positive performance in various indicators during the COVID-19 pandemic, the socioeconomic and cultural effects of COVID-19 have affected people’s health behaviour. In addition to social efforts to reduce the spread of infectious diseases, active measures to positively change health behaviour are needed.

## Figures and Tables

**Figure f2-epih-44-e2022043:**
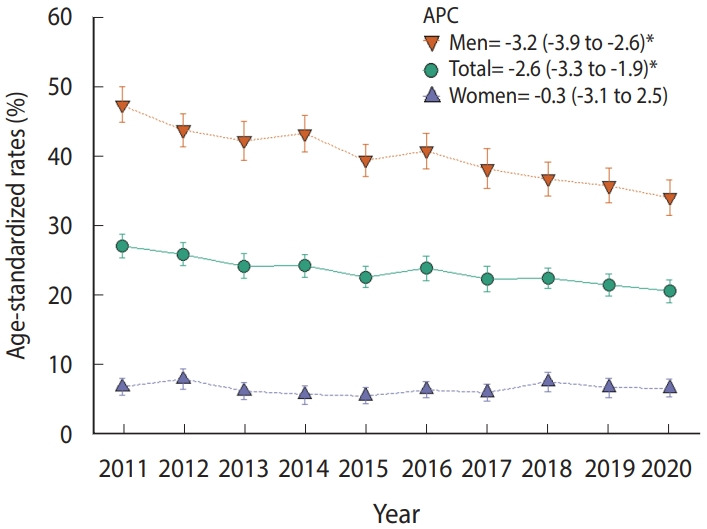


**Table 1.** Differences between periods and APCs in current cigarette smoking prevalence by demographic and socioeconomic indicators among Korean men and women aged 19 or older in the 2011-2020 KNHANES

Variables				Differences between, %p (95% CI)			APC (95% CI)
				2011-2019 and 2020	2017-2019 and 2020	2019 and 2020	
Men							
	Total			-6.7 (-9.4, -4.0)^*^	-2.9 (-5.8, 0.1)	-1.8 (-5.3, 1.8)	-3.2 (-3.9, -2.6)^*^
	Age (yr)		19-29	-6.7 (-11.8, -1.6)^*^	-4.7 (-10.4, 1.1)	-5.8 (-13.3, 1.7)	-2.4 (-4.2, -0.6)^*^
			30-39	-13.9 (-19.9, -8.0)^*^	-4.8 (-11.3, 1.7)	-3.4 (-11.2, 4.4)	-5.6 (-6.9, -4.2)^*^
			40-49	-4.7 (-10.2, 0.9)	-1.3 (-7.3, 4.7)	3.6 (-3.6, 10.8)	-2.3 (-4.1, -0.4)^*^
			50-59	-2.9 (-8.1, 2.4)	-1.6 (-7.4, 4.1)	-0.2 (-7.1, 6.7)	-1.7 (-2.8, -0.6)^*^
			60-69	-1.6 (-6.7, 3.5)	-0.5 (-6.0, 5.0)	-2.7 (-9.7, 4.2)	-1.9 (-4.8, 1.2)
			≥70	-3.7 (-8.3, 0.9)	-1.6 (-6.6, 3.4)	-2.0 (-7.9, 3.9)	-5.4 (-8.8, -1.8)^*^
	No. of household members		1	-4.3 (-11.3, 2.8)	-3.0 (-10.6, 4.7)	-0.6 (-10.0, 8.8)	-
			≥2	-7.5 (-10.3, -4.6)^*^	-3.1 (-6.2, 0.0)	-2.1 (-5.9, 1.7)	-3.6 (-4.3, -2.9)^*^
	Residential area		Urban areas	-5.8 (-8.7, -2.8)^*^	-2.0 (-5.3, 1.2)	-1.4 (-5.3, 2.5)	-3.1 (-3.6, -2.5)^*^
			Rural areas	-11.9 (-18.6, -5.2)^*^	-7.7 (-15.1, -0.2)^*^	-2.6 (-10.9, 5.7)	-4.1 (-6.1, -2.1)^*^
	Income		Lowest	-10.1 (-15.5, -4.7)^*^	-6.9 (-12.8, -1.0)^*^	-7.7 (-14.7, -0.7)^*^	-3.5 (-4.9, -2.1)^*^
			Lower middle	-5.7 (-11.3, -0.2)^*^	-3.3 (-9.3, 2.8)	1.0 (-6.5, 8.6)	-2.8 (-4.3, -1.2)^*^
			Middle	-6.9 (-12.7, -1.0)^*^	-2.9 (-9.2, 3.4)	-4.2 (-11.9, 3.5)	-3.0 (-4.6, -1.5)^*^
			Upper middle	-6.9 (-12.5, -1.4)^*^	-4.0 (-10.0, 2.0)	-3.7 (-10.9, 3.5)	-2.1 (-3.8, -0.3)^*^
			Highest	-3.2 (-9.4, 3.0)	3.2 (-3.4, 9.9)	5.6 (-2.1, 13.3)	-4.6 (-6.5, -2.7)^*^
	Education						
		Age (yr)					
		30-59	≤High school	-6.1 (-12.0, -0.1)^*^	-4.5 (-10.9, 1.9)	-0.3 (-7.8, 7.3)	-1.4 (-2.3, -0.5)^*^
			≥College	-8.4 (-12.5, -4.4)^*^	-3.0 (-7.5, 1.4)	-1.6 (-6.9, 3.8)	-4.5 (-6.4, -2.7)^*^
		≥60	≤Middle school	-2.0 (-8.1, 4.0)	-0.5 (-6.9, 5.9)	-1.3 (-8.9, 6.4)	-3.3 (-6.1, -0.5)^*^
			≥High school	-2.0 (-6.5, 2.6)	-0.7 (-5.6, 4.2)	-2.7 (-8.9, 3.5)	-1.5 (-4.8, 1.9)
	Occupation		Non-manual	-9.2 (-14.0, -4.5)^*^	-3.0 (-8.1, 2.1)	-0.7 (-6.8, 5.5)	-5.2 (-7.4, -3.0)^*^
			Manual	-6.3 (-11.6, -1.0)^*^	-3.6 (-9.4, 2.3)	1.4 (-5.7, 8.6)	-2.1 (-3.2, -1.0)^*^
			Others	-10.7 (-21.6, 0.2)	-7.4 (-19.3, 4.5)	-12.1 (-26.2, 2.0)	-
Woman							
	Total			0.1 (-1.2, 1.5)	-0.1 (-1.6, 1.5)	0.0 (-2.0, 2.0)	-0.3 (-3.1, 2.5)
	Age (yr)		19-29	1.2 (-2.8, 5.2)	0.6 (-3.7, 5.0)	0.7 (-4.8, 6.2)	-0.7 (-5.8, 4.7)
			30-39	1.2 (-2.2, 4.6)	1.4 (-2.3, 5.1)	1.6 (-2.8, 6.0)	-0.9 (-3.9, 2.1)
			40-49	-0.1 (-2.7, 2.4)	-1.3 (-4.2, 1.6)	-0.8 (-4.4, 2.7)	4.3 (-0.7, 9.6)
			50-59	-2.9 (-4.2, -1.6)^*^	-2.2 (-3.7, -0.7)^*^	-2.2 (-4.3, -0.1)^*^	-5.5 (-14.7, 4.6)
			60-69	0.9 (-1.4, 3.2)	0.6 (-1.9, 3.1)	-0.1 (-3.5, 3.2)	3.7 (-3.2, 11.0)
			≥70	-1.3 (-2.5, -0.1)^*^	-0.2 (-1.4, 1.0)	-0.8 (-2.4, 0.8)	-9.8 (-15.7, -3.6)^*^
	No. of household members		1	1.6 (-4.3, 7.4)	-0.5 (-7.1, 6.1)	-	-
			≥2	0.0 (-1.3, 1.3)	0.1 (-1.4, 1.6)	0.6 (-1.3, 2.5)	-1.3 (-4.0, 1.5)
	Residential area		Urban	0.6 (-0.9, 2.2)	0.5 (-1.2, 2.1)	0.2 (-2.0, 2.4)	-0.2 (-3.2, 2.8)
			Rural	-3.6 (-5.8, -1.4)^*^	-4.1 (-7.3, -1.0)^*^	-1.8 (-5.1, 1.4)	-0.9 (-14.6, 15.0)
	Income		Lowest	1.5 (-2.3, 5.3)	1.2 (-2.9, 5.2)	-0.9 (-6.0, 4.2)	0.9 (-2.5, 4.4)
			Lower middle	1.4 (-1.8, 4.6)	1.6 (-2.0, 5.2)	2.6 (-1.7, 7.0)	-0.7 (-6.7, 5.6)
			Middle	-0.6 (-3.1, 2.0)	-1.9 (-4.7, 0.9)	-2.6 (-6.3, 1.1)	3.6 (-1.2, 8.7)
			Upper middle	0.4 (-2.2, 3.0)	0.0 (-2.8, 2.8)	0.3 (-2.8, 3.4)	2.5 (0.4, 4.7)
			Highest	-1.4 (-2.8, 0.1)	-0.4 (-2.0, 1.2)	0.6 (-1.2, 2.4)	-7.1 (-14.1, 0.5)
	Education						
		Age (yr)					
		30-59	≤High school	2.9 (-1.4, 7.3)	0.4 (-4.4, 5.2)	-0.3 (-6.3, 5.7)	5.4 (1.2, 9.8)^*^
			≥College	-0.5 (-2.0, 0.9)	-0.6 (-2.1, 1.0)	0.0 (-1.9, 1.8)	-2.4 (-9.6, 5.5)
		≥60	≤Middle school	0.3 (-1.8, 2.5)	0.6 (-1.6, 2.9)	-0.3 (-3.4, 2.7)	-1.8 (-10.2, 7.4)
			≥High school	-1.2 (-2.8, 0.3)	-0.9 (-2.5, 0.7)	-1.3 (-3.3, 0.8)	-
	Occupation		Non-manual	1.2 (-1.2, 3.5)	0.8 (-1.8, 3.4)	0.6 (-2.5, 3.7)	4.1 (-0.7, 9.0)
			Manual	-2.7 (-7.1, 1.7)	-4.7 (-10.0, 0.5)	-4.8 (-11.9, 2.4)	1.2 (-4.0, 6.7)
			Others	0.5 (-2.0, 2.9)	0.7 (-1.9, 3.3)	1.7 (-1.3, 4.7)	-0.2 (-4.5, 4.4)

APC, annual percentage chanage; KNHANES, Korea National Health and Nutrition Examination Survey; CI, confidence interval.

* p<0.05.

**Table 2.** Differences between periods and APCs in high-risk drinking prevalence by demographic and socioeconomic indicators among Korean men and women aged 19 or older in the 2011-2020 KNHANES

Variables				Differences between, %p (95% CI)			APC (95% CI)
				2011-2019 and 2020	2017-2019 and 2020	2019 and 2020	
Men							
	Total			0.7 (-1.4, 2.8)	1.5 (-0.8, 3.8)	3.1 (0.3, 5.9)^*^	-0.7 (-2.1, 0.7)
	Age (yr)		19-29	-2.5 (-7.0, 2.0)	-1.3 (-6.2, 3.6)	1.2 (-4.7, 7.1)	-3.3 (-5.6, -1.0)^*^
			30-39	-0.9 (-5.9, 4.1)	2.1 (-3.2, 7.5)	4.4 (-2.2, 10.9)	-3.3 (-5.3, -1.2)^*^
			40-49	3.5 (-1.9, 8.9)	4.0 (-1.7, 9.8)	6.9 (0.4, 13.4)^*^	-0.2 (-2.1, 1.8)
			50-59	0.3 (-4.2, 4.8)	0.4 (-4.4, 5.3)	0.3 (-5.7, 6.3)	-0.1 (-1.8, 1.6)
			60-69	5.6 (1.5, 9.7)	3.4 (-1.1, 7.9)	3.5 (-1.6, 8.7)	6.5 (2.8, 10.4)^*^
			≥70	1.2 (-1.7, 4.0)	-0.4 (-3.6, 2.7)	-2.6 (-6.8, 1.6)	7.0 (2.2, 12.0)^*^
	No. of household members		1	-3.0 (-8.3, 2.3)	-1.6 (-7.3, 4.2)	-4.9 (-13.0, 3.2)	-
			≥2	1.1 (-1.2, 3.5)	1.9 (-0.7, 4.4)	3.9 (0.8, 6.9)^*^	-0.6 (-2.2, 1.1)
	Residential area		Urban	0.7 (-1.6, 3.0)	1.2 (-1.2, 3.7)	3.4 (0.3, 6.5)^*^	-0.7 (-2.1, 0.8)
			Rural	0.8 (-4.5, 6.2)	3.2 (-2.4, 8.7)	1.1 (-5.2, 7.3)	-0.7 (-4.7, 3.5)
	Income		Lowest	-2.4 (-6.4, 1.6)	-1.1 (-5.5, 3.3)	0.2 (-5.0, 5.3)	-2.1 (-4.4, 0.2)
			Lower middle	-0.7 (-5.1, 3.7)	-1.8 (-6.6, 3.1)	-2.7 (-9.0, 3.5)	0.2 (-3.0, 3.5)
			Middle	4.0 (-0.9, 8.9)	4.6 (-0.7, 9.9)	6.9 (0.9, 12.9)^*^	-1.0 (-4.9, 3.2)
			Upper middle	-1.5 (-6.7, 3.6)	-0.4 (-5.9, 5.1)	2.7 (-3.5, 9.0)	0.0 (-3.7, 3.9)
			Highest	4.0 (-0.7, 8.8)	6.3 (1.1, 11.5)	7.8 (1.4, 14.2)^*^	-1.1 (-4.1, 1.9)
	Education						
		Age (yr)					
		30-59	≤High school	3.7 (-1.6, 9.0)	4.1 (-1.7, 9.9)	9.4 (2.8, 16.0)^*^	0.1 (-2.7, 2.9)
			≥College	0.0 (-3.8, 3.9)	1.7 (-2.4, 5.8)	2.3 (-2.6, 7.2)	-1.8 (-3.5, -0.2)^*^
		≥60	≤Middle school	0.9 (-3.0, 4.8)	-1.5 (-6.0, 2.9)	-0.9 (-6.0, 4.1)	4.7 (0.4, 9.2)^*^
			≥High school	4.1 (0.6, 7.6)^*^	2.0 (-1.8, 5.9)	0.4 (-4.4, 5.2)	9.7 (6.1, 13.4)^*^
	Occupation		Non-manual	-2.4 (-6.6, 1.8)	0.2 (-4.3, 4.6)	2.0 (-3.8, 7.8)	-3.0 (-4.3, -1.6)^*^
			Manual	4.4 (-0.8, 9.6)	4.7 (-0.9, 10.3)	6.6 (0.0, 13.1)^*^	0.0 (-2.0, 2.2)
			Others	2.8 (-6.8, 12.4)	4.1 (-6.1, 14.4)	7.8 (-3.9, 19.6)	-
Woman							
	Total			-0.1 (-1.3, 1.1)	-1.1 (-2.5, 0.3)	-0.3 (-1.9, 1.4)	3.5 (0.3, 6.8)^*^
	Age (yr)		19-29	-1.7 (-5.2, 1.8)	-3.3 (-7.3, 0.7)	-0.6 (-5.1, 3.8)	2.8 (-2.7, 8.7)
			30-39	0.9 (-2.2, 4.1)	0.2 (-3.2, 3.6)	1.0 (-3.1, 5.0)	2.5 (-0.6, 5.6)
			40-49	1.2 (-1.2, 3.7)	0.0 (-2.6, 2.7)	0.0 (-3.4, 3.5)	4.8 (0.2, 9.6)^*^
			50-59	-0.8 (-2.4, 0.7)	-1.4 (-3.1, 0.4)	-0.5 (-2.6, 1.6)	2.3 (-4.1, 9.2)
			60-69	-0.8 (-1.7, 0.1)	-2.0 (-3.3, -0.8)^*^	-2.4 (-4.4, -0.5)^*^	14.9 (-0.5-32.7)
			≥70	-0.2 (-0.6, 0.3)	-0.2 (-0.7, 0.3)	-0.5 (-1.3, 0.3)	-
	No. of household members		1	3.2 (-3.2, 9.5)	3.2 (-3.6, 10.0)	-	-
			≥2	-0.4 (-1.6, 0.8)	-1.4 (-2.7, 0.0)	-0.2 (-1.8, 1.4)	3.2 (-0.6, 7.1)
	Residential area		Urban	0.1 (-1.2, 1.5)	-0.7 (-2.2, 0.8)	0.1 (-1.7, 1.9)	3.1 (0.3, 6.0)
			Rural	-1.8 (-4.7, 1.2)	-4.1 (-8.1, -0.2)^*^	-2.5 (-6.9, 1.9)	2.6 (-6.2, 12.3)
	Income		Lowest	0.7 (-2.8, 4.1)	-1.1 (-4.8, 2.7)	-2.2 (-6.9, 2.5)	4.6 (-0.8, 10.3)
			Lower middle	1.3 (-1.6, 4.1)	0.1 (-3.0, 3.3)	2.2 (-1.3, 5.8)	5.3 (-1.2, 12.2)
			Middle	-0.3 (-3.0, -2.4)	-1.9 (-5.0, 1.1)	-1.4 (-5.2, 2.4)	6.6 (-0.3, 13.9)
			Upper middle	0.0 (-2.5, 2.5)	-0.5 (-3.3, 2.4)	1.4 (-1.7, 4.4)	0.5 (-5.4, 6.7)
			Highest	-1.7 (-3.6, 0.2)	-1.9 (-4.1, 0.3)	-1.5 (-4.4, 1.4)	-0.2 (-4.6, 4.5)
	Education						
		Age (yr)					
		30-59	≤High school	1.8 (-1.9, 5.4)	-1.5 (-5.5, 2.5)	-1.6 (-6.7, 3.5)	7.5 (3.4, 11.8)^*^
			≥College	0.4 (-1.1, 2.0)	0.0 (-1.7, 1.7)	0.3 (-1.7, 2.3)	4.0 (-1.0, 9.2)
		≥60	≤Middle school	-0.8 (-1.5, -0.2)^*^	-1.8 (-2.7, -0.8)^*^	-2.1 (-3.7, -0.5)^*^	13.7 (-1.8, 31.6)
			≥High school	-0.4 (-1.1, 0.3)	-0.9 (-1.8, 0.1)	-1.4 (-2.9, 0.1)	-
	Occupation		Non-manual	1.6 (-0.7, 4.0)	0.9 (-1.7, 3.5)	1.5 (-1.4, 4.5)	6.7 (1.8, 11.9)^*^
			Manual	-3.3 (-7.3, 0.7)	-5.1 (-9.7, -0.5)^*^	-5.9 (-11.8, 0.0)	2.0 (-4.8, 9.3)
			Others	1.9 (-0.9, 4.6)	0.8 (-2.2, 3.8)	1.8 (-1.8, 5.4)	5.6 (0.2, 11.2)^*^

APC, annual percentage chanage; KNHANES, Korea National Health and Nutrition Examination Survey; CI, confidence interval.

* p<0.05.

**Table 3.** Differences between periods and APCs in the prevalence of inadequate physical activity by demographic and socioeconomic indicators among Korean men and women aged 19 or older in the 2014-2020 KNHANES

Variables				Differences between, %p (95% CI)			APC (95% CI)
				2014-2019 and 2020	2017-2019 and 2020	2019 and 2020	
Men							
	Total			5.5 (2.6-8.5)^*^	3.1 (0.0-6.2)^*^	4.3 (0.4, 8.1)^*^	3.8 (0.8, 6.8)^*^
	Age (yr)		19-29	8.8 (2.4, 15.1)^*^	6.6 (-0.2, 13.4)	6.7 (-1.5, 14.8)	6.6 (0.5, 13.1)^*^
			30-39	5.7 (-0.7, 12.2)	6.1 (-0.9, 13.0)	9.5 (1.2, 17.9)^*^	1.3 (-2.9, 5.6)
			40-49	7.3 (2.1, 12.4)^*^	4.4 (-1.2, 9.9)	3.8 (-3.0, 10.6)	4.1 (2.5, 5.6)^*^
			50-59	5.4 (-0.9, 11.7)	0.8 (-5.9, 7.4)	0.6 (-7.3, 8.5)	5.1 (0.4, 10.0)^*^
			60-69	0.3 (-5.4, 6.0)	-4.3 (-10.3, 1.8)	0.0 (-7.3, 7.3)	2.2 (-3.6, 8.4)
			≥70	-3.1 (-9.5, 3.2)	-7.1 (-13.8, -0.4)^*^	-5.9 (-13.9, 2.0)	2.6 (-2.0, 7.4)
	No. of household members		1	6.4 (-1.7, 14.4)	2.7 (-5.8, 11.1)	1.0 (-10.0, 12.0)	-
			≥2	5.5 (2.3, 8.7)^*^	3.1 (-0.2, 6.5)	4.5 (0.4, 8.7)^*^	3.6 (0.5-6.8)^*^
	Residential area		Urban	5.6 (2.5, 8.8)^*^	2.8 (-0.6, 6.2)	4.4 (0.3, 8.5)^*^	4.2 (0.9, 7.6)^*^
			Rural	6.1 (-2.1, 14.2)	5.2 (-3.6, 14.0)	3.8 (-7.1, 14.7)	2.8 (-0.5, 6.2)
	Income		Lowest	6.5 (0.1, 12.8)^*^	4.8 (-1.8, 11.5)	4.4 (-3.9, 12.6)	3.5 (-1.6, 8.9)
			Lower middle	5.8 (-0.1, 11.7)	2.6 (-3.7, 8.9)	4.1 (-3.6, 11.9)	4.1 (1.3, 6.9)^*^
			Middle	4.4 (-1.5, 10.3)	1.6 (-4.6, 7.8)	3.0 (-4.6, 10.5)	3.6 (-0.2, 7.5)
			Upper middle	7.9 (1.5, 14.2)^*^	6.5 (-0.1, 13.2)	9.9 (2.3, 17.6)^*^	2.9 (-2.8, 9.0)
			Highest	3.9 (-2.8, 10.5)	0.8 (-6.1, 7.7)	0.4 (-7.6, 8.3)	3.3 (-0.2, 6.9)
	Education						
		Age (yr)					
		30-59	≤High school	5.3 (-0.3, 10.9)	2.1 (-4.0, 8.2)	5.2 (-3.0, 13.4)	3.3 (-0.6, 7.4)
			≥College	7.4 (2.5, 12.3)^*^	5.5 (0.4, 10.7)^*^	5.7 (-0.4, 11.8)	3.8 (2.1, 5.5)^*^
		≥60	≤Middle school	6.1 (0.8, 11.5)^*^	1.0 (-4.7, 6.7)	3.7 (-3.5, 10.9)	3.5 (0.2, 6.9)^*^
			≥High school	-4.6 (-10.4, 1.2)	-8.5 (-14.8, -2.2)^*^	-5.7 (-13.3, 2.0)	1.3 (-5.1, 8.3)
	Occupation		Non-manual	6.1 (0.2, 12.0)^*^	3.9 (-2.2, 10.1)	4.3 (-2.8, 11.4)	3.4 (1.1, 5.8)^*^
			Manual	5.4 (0.0, 10.8)^*^	3.5 (-2.4, 9.4)	3.9 (-3.7, 11.4)	2.9 (-0.6, 6.5)
			Others	10.8 (0.0, 21.6)	8.7 (-2.9, 20.2)	9.9 (-4.2, 24.0)	-
Woman							
	Total			4.3 (1.6, 6.9)^*^	1.5 (-1.4, 4.3)	-0.2 (-3.9, 3.4)	3.3 (1.5, 5.1)^*^
	Age (yr)		19-29	6.0 (-0.3, 12.3)	5.1 (-1.7, 12.0)	1.6 (-7.2, 10.3)	3.6 (0.2, 7.2)^*^
			30-39	2.0 (-4.1, 8.1)	-2.2 (-8.7, 4.2)	-6.1 (-13.8, 1.6)	4.0 (0.1, 8.1)^*^
			40-49	6.2 (1.3, 11.1)^*^	2.4 (-2.8, 7.7)	1.2 (-5.2, 7.7)	4.6 (1.6, 7.6)^*^
			50-59	4.8 (-0.2, 9.8)	1.9 (-3.4, 7.2)	2.4 (-4.1, 8.9)	3.1 (0.6, 5.6)^*^
			60-69	2.3 (-2.9, 7.5)	-0.8 (-6.4, 4.8)	-2.3 (-9.0, 4.5)	2.5 (0.7, 4.2)^*^
			≥70	2.2 (-2.4, 6.7)	1.3 (-3.5, 6.2)	6.3 (-0.1, 12.6)	0.8 (-1.3, 3.0)
	No. of household members		1	-0.9 (-10.6, 8.8)	-2.9 (-13.0, 7.3)	-	-
			≥2	4.6 (1.8, 7.4)^*^	1.7 (-1.3, 4.7)	0.1 (-3.7, 3.9)	3.5 (1.8, 5.1)^*^
	Residential area		Urban	3.9 (1.1, 6.7)^*^	1.1 (-1.8, 4.1)	-0.5 (-4.2, 3.2)	3.4 (1.3, 5.6)^*^
			Rural	7.9 (1.3, 14.5)^*^	5.0 (-2.1, 12.1)	3.9 (-6.3, 14.1)	3.5 (1.7, 5.3)^*^
	Income		Lowest	5.9 (0.2, 11.5)^*^	3.1 (-2.8, 9.0)	1.6 (-5.7, 8.9)	3.6 (1.6, 5.7)^*^
			Lower middle	3.1 (-2.6, 8.9)	0.4 (-5.7, 6.5)	-1.4 (-9.0, 6.2)	3.3 (-0.8, 7.5)
			Middle	2.6 (-3.3, 8.5)	0.1 (-6.2, 6.4)	-0.7 (-8.0, 6.7)	2.7 (1.1, 4.3)^*^
			Upper middle	2.7 (-2.9, 8.3)	-0.6 (-6.5, 5.3)	-2.2 (-9.4, 5.0)	3.1 (0.3, 5.9)^*^
			Highest	6.8 (1.7, 12.0)^*^	3.8 (-1.8, 9.3)	0.4 (-6.3, 7.1)	4.4 (2.8, 6.1)^*^
	Education						
		Age (yr)					
		30-59	≤High school	4.9 (-1.0, 10.8)	0.7 (-5.6, 7.0)	1.4 (-6.1, 9.0)	3.8 (1.0, 6.6)^*^
			≥College	4.0 (-0.4, 8.4)	0.1 (-4.6, 4.9)	-3.4 (-9.2, 2.4)	4.8 (1.5, 8.2)^*^
		≥60	≤Middle school	2.6 (-2.2, 7.4)	0.2 (-4.9, 5.2)	1.2 (-4.9, 7.3)	1.8 (0.3, 3.3)^*^
			≥High school	2.9 (-5.3, 11.1)	1.9 (-6.7, 10.4)	3.3 (-6.6, 13.2)	1.2 (-0.2, 2.6)
	Occupation		Non-manual	2.4 (-3.0, 7.9)	-0.4 (-6.1, 5.3)	-2.3 (-9.2, 4.7)	3.0 (0.7, 5.4)^*^
			Manual	0.8 (-6.2, 7.8)	-2.1 (-9.7, 5.4)	-1.0 (-10.0, 8.0)	2.2 (-0.4, 4.9)
			Others	7.3 (2.3, 12.3)^*^	2.7 (-2.7, 8.1)	-0.5 (-6.9, 5.9)	5.2 (2.5, 8.1)^*^

APC, annual percentage chanage; KNHANES, Korea National Health and Nutrition Examination Survey; CI, confidence interval.

* p<0.05.

## SUPPLEMENTARY MATERIALS

Supplementary Material 1. Numbers and age-standardized rates (%) of current cigarette smoking by demographic and socioeconomic indicators among Koreans (men and women combined) aged 19 or older in the 2011-2020 Korea National Health and Nutrition Examination Survey.

Supplementary Material 2 Numbers and age-standardized rates (%) of current cigarette smoking by demographic and socioeconomic indicators among Korean men aged 19 or older in the 2011-2020 Korea National Health and Nutrition Examination Survey.

Supplementary Material 3 Numbers and age-standardized rates (%) of current cigarette smoking by demographic and socioeconomic indicators among Korean women aged 19 or older in the 2011-2020 Korea National Health and Nutrition Examination Survey.

Supplementary Material 4 Numbers and age-standardized rates (%) of high-risk drinking by demographic and socioeconomic indicators among Koreans (men and women combined) aged 19 or older in the 2011-2020 Korea National Health and Nutrition Examination Survey.

Supplementary Material 5 Numbers and age-standardized rates (%) of high-risk drinking by demographic and socioeconomic indicators among Korean men aged 19 or older in the 2011-2020 Korea National Health and Nutrition Examination Survey.

Supplementary Material 6 Numbers and age-standardized rates (%) of high-risk drinking by demographic and socioeconomic indicators among Korean women aged 19 or older in the 2011-2020 Korea National Health and Nutrition Examination Survey.

Supplementary Material 7 Numbers and age-standardized rates (%) of inadequate physical activity by demographic and socioeconomic indicators among Koreans (men and women combined) aged 19 or older in the 2014-2020 Korea National Health and Nutrition Examination Survey.

Supplementary Material 8 Numbers and age-standardized rates (%) of inadequate physical activity by demographic and socioeconomic indicators among Korean men aged 19 or older in the 2014-2020 Korea National Health and Nutrition Examination Survey.

Supplementary Material 9 Numbers and age-standardized rates (%) of inadequate physical activity by demographic and socioeconomic indicators among Korean women aged 19 or older in the 2014-2020 Korea National Health and Nutrition Examination Survey.

Supplementary Material 10 Differences between periods and annual percentage changes of current cigarette smoking by demographic and socioeconomic indicators among Koreans (men and women combined) aged 19 or older in the 2011-2020 Korea National Health and Nutrition Examination Survey.

Supplementary Material 11 Differences between periods and annual percentage changes of high-risk drinking by demographic and socioeconomic indicators among Koreans (men and women combined) aged 19 or older in the 2011-2020 Korea National Health and Nutrition Examination Survey.

Supplementary Material 12 Differences between periods and annual percentage changes of inadequate physical activity by demographic and socioeconomic indicators among Koreans (men and women combined) aged 19 or older in the 2011-2020 Korea National Health and Nutrition Examination Survey.
