# Supplementary material for: Smoking, drinking, and physical activity among Korean adults before and during the COVID-19 pandemic: a special report of the 2020 Korea National Health and Nutrition Examination Survey
Source: Epidemiol Health. 2022 Apr 25;44:e2022043. doi: 10.4178/epih.e2022043 (PMC9133597; doi:10.4178/epih.e2022043)
Supplement: Supplementary Material 1. — Numbers and age-standardized rates (%) of current cigarette smoking by demographic and socioeconomic indicators among Koreans (men and women combined) aged 19 or older in the 2011-2020 Korea National Health and Nutrition Examination Survey. [file epih-44-e2022043-suppl1.docx]

Supplementary Material 1. Numbers and age-standardized rates (%) of current cigarette smoking by demographic and socioeconomic indicators among Koreans (men and women combined) aged 19 or older in the 2011-2020 Korea National Health and Nutrition Examination Survey.

|  |  | 2011 | 2012 | 2013 | 2014 | 2015 | 2016 | 2017 | 2018 | 2019 | 2020 |
| --- | --- | --- | --- | --- | --- | --- | --- | --- | --- | --- | --- |
| Total |  | 6,023  27.1 (25.4-28.8) | 5,591  25.8 (24.2-27.5) | 5,338  24.1 (22.4-25.9) | 5,192  24.2 (22.6-25.8) | 5,405  22.6 (21.1-24.1) | 6,015  23.9 (22.1-25.6) | 6,115  22.3 (20.5-24.1) | 6,183  22.4 (20.9-23.9) | 6,190  21.5 (19.9-23.0) | 5,858  20.6 (18.9-22.2) |
| Age | 19-29 | 676  28.3 (23.6-33.0) | 637  28.0 (23.5-32.5) | 701  24.1 (19.9-28.2) | 594  22.5 (17.8-27.1) | 682  23.7 (19.9-27.5) | 695  25.4 (21.1-29.7) | 724  24.4 (20.5-28.4) | 762  23.8 (19.9-27.6) | 749  24.8 (20.8-28.8) | 798  21.9 (18.7-25.1) |
|  | 30-39 | 1,097  36.6 (33.3-39.9) | 960  32.5 (28.8-36.2) | 938  30.7 (27.0-34.4) | 904  30.0 (26.3-33.7) | 744  27.7 (23.9-31.4) | 1,078  30.4 (26.9-34.0) | 904  25.5 (21.7-29.2) | 909  24.7 (21.4-28.0) | 915  24.0 (20.6-27.4) | 766  23.0 (19.4-26.6) |
|  | 40-49 | 1,056  25.7 (22.7-28.8) | 958  27.7 (24.3-31.1) | 1,040  26.9 (23.8-29.9) | 901  29.2 (25.9-32.6) | 951  25.4 (21.9-28.9) | 1,124  25.0 (21.8-28.2) | 1,122  26.3 (23.3-29.2) | 1,127  26.7 (23.8-29.7) | 1,107  22.4 (19.4-25.4) | 965  24.0 (20.7-27.3) |
|  | 50-59 | 1,184  24.5 (21.3-27.8) | 1,067  24.6 (21.5-27.6) | 1,013  22.0 (18.8-25.2) | 995  20.6 (17.6-23.7) | 1,131  20.8 (17.8-23.8) | 1,092  22.7 (19.6-25.7) | 1,204  20.2 (17.2-23.2) | 1,187  22.8 (19.8-25.8) | 1,167  20.3 (17.6-23.0) | 1,060  19.2 (16.5-21.9) |
|  | 60-69 | 1,023  17.5 (14.3-20.7) | 1,004  13.4 (10.7-16.2) | 847  17.4 (14.5-20.2) | 919  18.2 (15.5-21.0) | 992  14.1 (11.6-16.5) | 998  14.6 (12.1-17.1) | 1,089  14.4 (12.0-16.8) | 1,100  14.9 (12.2-17.6) | 1,112  16.8 (13.8-19.9) | 1,113  15.5 (12.8-18.3) |
|  | 70+ | 987  14.3 (11.0-17.5) | 965  10.9 (8.4-13.4) | 799  8.0 (6.0-9.9) | 879  10.1 (7.9-12.4) | 905  9.0 (6.8-11.2) | 1,028  9.1 (6.8-11.4) | 1,072  8.5 (6.6-10.3) | 1,098  6.6 (4.8-8.5) | 1,140  8.4 (6.6-10.2) | 1,156  7.2 (5.3-9.1) |
| Number of household members | 1 | 446  44.2 (34.7-53.7) | 486  37.7 (30.7-44.7) | 498  39.4 (31.7-47.1) | 518  38.7 (31.1-46.4) | 567  33.9 (26.7-41.0) | 676  31.0 (25.8-36.1) | 807  36.4 (30.9-41.8) | 799  36.6 (30.5-42.7) | 824  37.4 (32.2-42.7) | 802  36.6 (31.1-42.1) |
|  | 2+ | 5,577  26.4 (24.7-28.0) | 5,105  25.2 (23.5-26.9) | 4,839  23.3 (21.6-25.0) | 4,674  23.3 (21.8-24.9) | 4,838  21.9 (20.4-23.5) | 5,339  23.2 (21.4-25.0) | 5,308  21.0 (19.2-22.8) | 5,384  21.0 (19.5-22.5) | 5,366  20.0 (18.3-21.6) | 5,056  19.0 (17.4-20.6) |
| Residential area | Urban areas | 4,809  26.2 (24.3-28.0) | 4,466  25.4 (23.6-27.2) | 4,307  23.3 (21.4-25.3) | 4,192  23.4 (21.7-25.0) | 4,364  22.4 (20.7-24.1) | 4,853  23.1 (21.3-24.9) | 4,991  21.5 (19.7-23.3) | 5,060  21.7 (20.1-23.3) | 4,958  21.3 (19.6-23.0) | 4,661  20.7 (18.9-22.5) |
|  | Rural areas | 1,214  31.6 (26.8-36.4) | 1,125  28.2 (23.7-32.6) | 1,031  27.5 (23.4-31.7) | 1,000  29.7 (25.7-33.8) | 1,041  23.6 (19.3-27.9) | 1,162  28.8 (23.6-34.1) | 1,124  27.5 (22.2-32.8) | 1,123  27.3 (23.8-30.8) | 1,232  21.5 (18.0-24.9) | 1,197  19.2 (15.3-23.1) |
| Income | Lowest | 1,204  32.9 (29.6-36.3) | 1,079  29.6 (25.8-33.4) | 1,036  28.9 (25.4-32.3) | 1,010  28.1 (24.7-31.6) | 1,045  26.0 (22.5-29.6) | 1,192  25.6 (22.0-29.2) | 1,211  26.0 (22.1-29.9) | 1,234  25.1 (22.1-28.2) | 1,236  27.9 (24.7-31.1) | 1,149  23.7 (20.1-27.3) |
|  | Lower middle | 1,184  29.1 (25.7-32.5) | 1,116  28.4 (24.9-31.9) | 1,052  26.5 (22.9-30.2) | 1,037  24.3 (21.1-27.5) | 1,076  21.6 (18.2-24.9) | 1,201  26.0 (22.8-29.2) | 1,215  24.9 (21.4-28.5) | 1,236  26.7 (23.2-30.1) | 1,222  21.8 (18.5-25.1) | 1,162  23.6 (20.3-26.8) |
|  | Middle | 1,190  26.0 (22.6-29.4) | 1,087  23.5 (19.7-27.2) | 1,080  24.0 (20.6-27.4) | 1,036  25.8 (22.3-29.3) | 1,081  24.2 (20.4-27.9) | 1,201  22.5 (18.9-26.0) | 1,221  22.7 (19.5-25.9) | 1,242  21.6 (18.2-25.1) | 1,225  23.7 (20.4-27.1) | 1,172  19.6 (16.2-23.0) |
|  | Upper middle | 1,191  22.7 (19.4-25.9) | 1,110  22.5 (19.0-26.0) | 1,063  20.9 (17.9-24.0) | 1,051  23.1 (19.3-26.9) | 1,084  21.7 (18.2-25.3) | 1,203  23.5 (19.9-27.2) | 1,228  21.7 (18.5-25.0) | 1,222  20.5 (17.5-23.6) | 1,244  20.5 (17.2-23.8) | 1,176  18.9 (15.7-22.1) |
|  | Highest | 1,206  23.6 (20.4-26.9) | 1,134  23.8 (20.2-27.5) | 1,079  20.7 (17.5-23.9) | 1,039  19.7 (16.7-22.7) | 1,088  18.9 (15.6-22.3) | 1,199  21.7 (18.2-25.2) | 1,220  15.9 (13.1-18.8) | 1,231  17.2 (14.4-20.0) | 1,236  14.0 (11.4-16.6) | 1,179  17.4 (14.0-20.8) |
| Education  (aged 30-59 years) | ≤High school | 1,979  32.4 (29.6-35.1) | 1,751  30.5 (27.3-33.6) | 1,742  30.0 (27.1-32.9) | 1,499  29.9 (26.6-33.1) | 1,454  30.2 (26.3-34.0) | 1,553  29.9 (26.5-33.3) | 1,451  32.2 (28.7-35.7) | 1,483  33.5 (30.1-37.0) | 1,380  31.0 (27.5-34.5) | 1,184  31.0 (27.1-35.0) |
|  | ≥College | 1,347  28.0 (25.1-31.0) | 1,230  27.4 (24.1-30.7) | 1,247  25.5 (22.3-28.7) | 1,157  27.6 (24.8-30.4) | 1,164  20.4 (17.6-23.1) | 1,611  23.9 (21.6-26.1) | 1,609  20.6 (17.7-23.4) | 1,619  20.2 (17.7-22.6) | 1,706  18.6 (16.3-20.9) | 1,477  17.5 (15.3-19.8) |
| Education  (aged ≥60 years) | ≤Middle school | 1,503  15.2 (12.9-17.4) | 1,437  12.4 (10.2-14.5) | 1,227  12.2 (10.3-14.2) | 1,198  13.3 (11.0-15.6) | 1,206  9.0 (7.1-11.0) | 1,374  11.3 (9.1-13.5) | 1,412  11.3 (9.3-13.4) | 1,404  10.1 (7.9-12.3) | 1,368  12.0 (9.6-14.4) | 1,218  11.2 (8.6-13.8) |
|  | ≥ High school | 505  20.0 (14.3-25.7) | 527  12.9 (9.8-15.9) | 419  16.1 (12.1-20.1) | 497  17.5 (13.1-21.9) | 539  15.2 (11.4-19.1) | 574  13.7 (10.2-17.2) | 634  12.4 (9.7-15.0) | 699  13.0 (9.9-16.0) | 734  14.3 (11.2-17.5) | 746  12.2 (9.3-15.0) |
| Occupation | Non-manual | 916  33.1 (29.4-36.8) | 885  29.7 (25.9-33.6) | 861  25.3 (21.6-28.9) | 836  31.8 (28.2-35.4) | 826  22.2 (19.4-25.1) | 1,071  26.4 (23.5-29.4) | 1,151  21.7 (18.9-24.5) | 1,143  21.5 (18.5-24.4) | 1,162  19.3 (16.3-22.2) | 1,006  19.1 (16.1-22.1) |
|  | Manual | 1,399  40.1 (36.8-43.4) | 1,207  36.6 (33.1-40.1) | 1,263  38.0 (34.5-41.4) | 1,058  37.5 (33.8-41.3) | 1,053  34.4 (30.4-38.5) | 1,204  36.8 (33.3-40.3) | 1,130  38.0 (33.9-42.1) | 1,239  37.0 (33.1-40.9) | 1,124  33.0 (29.2-36.8) | 964  32.9 (28.7-37.0) |
|  | Others | 1,011  12.7 (9.7-15.6) | 887  16.1 (12.0-20.2) | 867  13.5 (10.4-16.6) | 763  9.8 (7.2-12.3) | 735  13.6 (10.1-17.2) | 890  14.0 (11.4-16.7) | 780  11.8 (8.7-15.0) | 716  13.0 (9.9-16.1) | 794  14.0 (11.3-16.8) | 688  13.7 (10.7-16.7) |
