# Supplementary material for: Smoking, drinking, and physical activity among Korean adults before and during the COVID-19 pandemic: a special report of the 2020 Korea National Health and Nutrition Examination Survey
Source: Epidemiol Health. 2022 Apr 25;44:e2022043. doi: 10.4178/epih.e2022043 (PMC9133597; doi:10.4178/epih.e2022043)
Supplement: Supplementary Material 2 — Numbers and age-standardized rates (%) of current cigarette smoking by demographic and socioeconomic indicators among Korean men aged 19 or older in the 2011-2020 Korea National Health and Nutrition Examination Survey. [file epih-44-e2022043-suppl2.docx]

Supplementary Material 2. Numbers and age-standardized rates (%) of current cigarette smoking by demographic and socioeconomic indicators among Korean men aged 19 or older in the 2011-2020 Korea National Health and Nutrition Examination Survey.

|  |  | 2011 | 2012 | 2013 | 2014 | 2015 | 2016 | 2017 | 2018 | 2019 | 2020 |
| --- | --- | --- | --- | --- | --- | --- | --- | --- | --- | --- | --- |
| Total |  | 2,557  47.3 (44.8-49.9) | 2,309  43.7 (41.3-46.1) | 2,254  42.2 (39.4-44.9) | 2,154  43.2 (40.6-45.8) | 2,352  39.4 (37.0-41.7) | 2,592  40.7 (38.1-43.3) | 2,717  38.1 (35.3-41.0) | 2,713  36.7 (34.2-39.1) | 2,744  35.7 (33.3-38.2) | 2,627  34.0 (31.4-36.5) |
| Age | 19-29 | 286  44.9 (38.5-51.4) | 253  41.5 (34.7-48.4) | 312  37.0 (31.1-42.8) | 239  34.8 (27.9-41.7) | 335  38.7 (33.0-44.4) | 304  41.7 (35.1-48.3) | 344  37.3 (31.2-43.4) | 359  34.9 (28.8-41.1) | 388  37.8 (31.9-43.7) | 398  32.0 (27.3-36.7) |
|  | 30-39 | 443  63.7 (58.8-68.6) | 389  54.8 (49.6-60.0) | 386  54.5 (48.6-60.5) | 378  53.2 (47.9-58.5) | 299  48.0 (41.9-54.1) | 457  51.5 (46.6-56.4) | 405  42.7 (36.9-48.5) | 416  39.9 (34.7-45.0) | 423  39.2 (33.9-44.5) | 338  35.8 (30.2-41.4) |
|  | 40-49 | 452  47.0 (41.2-52.7) | 401  49.5 (44.0-55.0) | 446  48.0 (43.0-53.1) | 365  54.4 (48.6-60.1) | 397  45.8 (40.2-51.4) | 500  43.9 (38.5-49.3) | 496  46.3 (41.7-50.9) | 475  44.1 (39.2-49.0) | 483  37.9 (33.1-42.8) | 419  41.5 (36.2-46.9) |
|  | 50-59 | 508  44.4 (38.7-50.0) | 434  41.8 (36.0-47.5) | 415  40.8 (35.2-46.4) | 400  39.4 (33.8-45.0) | 485  36.5 (31.3-41.7) | 451  38.2 (32.9-43.6) | 541  37.0 (31.7-42.3) | 500  40.6 (35.5-45.7) | 477  36.7 (32.0-41.3) | 481  36.5 (31.5-41.4) |
|  | 60-69 | 460  32.5 (26.5-38.4) | 432  26.9 (21.6-32.2) | 378  32.5 (27.1-38.0) | 405  35.8 (30.7-40.9) | 460  26.1 (21.5-30.7) | 441  25.7 (21.2-30.2) | 469  26.6 (22.1-31.0) | 499  26.7 (22.1-31.3) | 487  30.1 (25.1-35.0) | 483  27.3 (22.4-32.2) |
|  | 70+ | 408  28.8 (22.7-34.9) | 400  23.2 (17.9-28.5) | 317  15.6 (11.3-19.8) | 367  19.4 (15.0-23.8) | 376  17.0 (12.2-21.9) | 439  18.0 (14.0-22.0) | 462  18.2 (13.9-22.4) | 464  14.7 (10.5-19.0) | 486  17.0 (13.1-20.9) | 508  15.0 (10.5-19.4) |
| Number of household members | 1 | 131  - - | 130  - - | 158  - - | 163  - - | 203  47.6 (37.9-57.3) | 252  46.4 (39.1-53.7) | 328  49.2 (43.2-55.2) | 310  50.8 (42.6-58.9) | 318  47.1 (40.2-54.1) | 343  46.5 (40.0-53.1) |
|  | 2+ | 2,426  46.8 (44.2-49.4) | 2,179  43.2 (40.7-45.7) | 2,095  41.3 (38.5-44.1) | 1,991  42.0 (39.5-44.5) | 2,149  38.6 (36.2-41.1) | 2,340  40.1 (37.4-42.8) | 2,389  36.7 (33.7-39.7) | 2,403  35.1 (32.7-37.5) | 2,426  34.4 (31.8-37.0) | 2,284  32.3 (29.6-35.0) |
| Residential area | Urban areas | 2,030  45.7 (42.9-48.5) | 1,826  43.3 (40.7-45.9) | 1,802  41.0 (37.9-44.1) | 1,717  42.1 (39.3-44.9) | 1,879  39.2 (36.6-41.8) | 2,087  39.8 (37.0-42.5) | 2,210  36.4 (33.4-39.3) | 2,208  36.1 (33.5-38.8) | 2,179  35.4 (32.8-38.1) | 2,082  34.0 (31.2-36.8) |
|  | Rural areas | 527  54.5 (48.3-60.7) | 483  45.4 (38.7-52.1) | 452  47.4 (40.2-54.6) | 437  50.2 (44.2-56.3) | 473  40.9 (34.0-47.8) | 505  47.2 (40.3-54.1) | 507  48.5 (40.0-57.0) | 505  39.1 (35.0-43.3) | 565  36.4 (31.0-41.8) | 545  33.8 (27.4-40.2) |
| Income | Lowest | 509  54.3 (48.9-59.8) | 444  48.7 (43.2-54.2) | 442  47.9 (42.6-53.1) | 419  46.3 (40.6-52.0) | 461  41.8 (36.1-47.5) | 521  40.3 (34.5-46.0) | 537  42.8 (37.3-48.4) | 540  40.1 (35.0-45.1) | 552  42.7 (37.9-47.5) | 517  34.9 (29.9-40.0) |
|  | Lower middle | 502  50.0 (44.0-55.9) | 452  48.0 (42.7-53.3) | 448  44.1 (38.5-49.7) | 434  42.7 (37.3-48.2) | 460  38.3 (33.3-43.4) | 518  44.0 (38.9-49.0) | 534  43.4 (37.9-48.9) | 540  41.8 (36.6-47.1) | 541  36.2 (30.8-41.7) | 525  37.3 (32.0-42.6) |
|  | Middle | 506  47.0 (41.1-52.9) | 448  39.7 (33.6-45.8) | 446  43.8 (38.3-49.2) | 431  46.4 (40.5-52.2) | 468  42.2 (36.4-47.9) | 507  39.8 (34.3-45.2) | 543  37.3 (32.2-42.3) | 549  35.4 (30.0-40.8) | 539  38.3 (32.9-43.6) | 522  34.1 (28.6-39.5) |
|  | Upper middle | 502  41.7 (36.4-47.1) | 462  39.5 (33.9-45.1) | 443  38.6 (33.2-44.0) | 433  42.7 (36.6-48.9) | 470  40.0 (34.0-45.9) | 515  43.4 (37.6-49.2) | 547  38.5 (33.4-43.7) | 538  34.4 (29.1-39.6) | 548  36.1 (31.1-41.2) | 525  32.4 (27.2-37.6) |
|  | Highest | 516  42.7 (37.5-48.0) | 476  41.2 (35.2-47.3) | 461  37.2 (32.0-42.4) | 434  37.6 (32.4-42.8) | 478  33.9 (28.9-39.0) | 522  36.5 (31.1-42.0) | 549  28.0 (23.1-32.8) | 538  31.0 (26.2-35.9) | 552  25.9 (21.1-30.7) | 532  31.5 (25.6-37.4) |
| Education  (aged 30-59 years) | ≤High school | 732  58.7 (54.2-63.1) | 629  55.8 (50.8-60.9) | 642  55.5 (51.0-60.0) | 537  56.3 (51.1-61.5) | 541  55.3 (50.3-60.4) | 565  53.0 (47.8-58.2) | 583  57.2 (52.1-62.4) | 562  54.0 (49.1-59.0) | 533  49.5 (44.6-54.4) | 486  49.2 (43.4-55.1) |
|  | ≥College | 663  46.7 (42.6-50.7) | 593  42.2 (37.6-46.8) | 604  42.9 (38.0-47.8) | 546  47.3 (43.0-51.6) | 532  35.3 (30.8-39.7) | 776  39.7 (36.3-43.0) | 767  34.7 (30.4-39.1) | 768  33.9 (29.8-38.1) | 792  32.1 (28.4-35.9) | 692  30.6 (26.8-34.3) |
| Education  (aged ≥60 years) | ≤Middle school | 513  34.8 (29.2-40.3) | 478  30.3 (25.7-34.9) | 415  26.7 (21.9-31.4) | 409  31.1 (26.0-36.2) | 422  21.6 (16.9-26.2) | 477  23.9 (19.6-28.2) | 481  27.0 (22.5-31.6) | 476  23.4 (18.4-28.3) | 472  26.3 (21.2-31.4) | 419  25.0 (19.1-31.0) |
|  | ≥ High school | 355  24.5 (18.0-31.0) | 352  17.6 (13.4-21.8) | 280  23.5 (18.0-29.0) | 317  26.8 (20.6-33.1) | 353  20.7 (15.6-25.9) | 371  19.7 (14.8-24.5) | 396  18.2 (14.0-22.4) | 438  19.3 (15.1-23.4) | 439  21.9 (17.3-26.4) | 448  19.2 (14.9-23.5) |
| Occupation | Non-manual | 551  47.6 (42.8-52.5) | 505  44.2 (39.1-49.3) | 468  39.4 (34.0-44.8) | 456  48.0 (43.2-52.8) | 428  35.8 (31.4-40.2) | 579  39.6 (35.8-43.5) | 601  34.1 (29.8-38.4) | 584  33.0 (28.6-37.4) | 587  30.2 (26.0-34.3) | 507  29.5 (25.0-34.0) |
|  | Manual | 737  57.1 (53.1-61.1) | 609  52.2 (47.3-57.0) | 664  54.3 (49.7-59.0) | 548  54.5 (49.8-59.3) | 534  48.7 (43.9-53.5) | 639  50.9 (46.5-55.3) | 626  52.3 (47.1-57.5) | 638  50.8 (45.9-55.7) | 609  44.2 (39.3-49.1) | 540  45.6 (40.5-50.7) |
|  | Others | 106  - - | 107  58.6 (46.6-70.5) | 115  52.0 (42.4-61.5) | 79  - - | 109  - - | 124  45.6 (36.5-54.8) | 124  42.9 (30.8-55.0) | 104  42.2 (32.4-52.1) | 124  50.3 (40.7-59.8) | 130  38.2 (27.9-48.5) |
