# Supplementary material for: Smoking, drinking, and physical activity among Korean adults before and during the COVID-19 pandemic: a special report of the 2020 Korea National Health and Nutrition Examination Survey
Source: Epidemiol Health. 2022 Apr 25;44:e2022043. doi: 10.4178/epih.e2022043 (PMC9133597; doi:10.4178/epih.e2022043)
Supplement: Supplementary Material 3 — Numbers and age-standardized rates (%) of current cigarette smoking by demographic and socioeconomic indicators among Korean women aged 19 or older in the 2011-2020 Korea National Health and Nutrition Examination Survey. [file epih-44-e2022043-suppl3.docx]

Supplementary Material 3. Numbers and age-standardized rates (%) of current cigarette smoking by demographic and socioeconomic indicators among Korean women aged 19 or older in the 2011-2020 Korea National Health and Nutrition Examination Survey.

|  |  | 2011 | 2012 | 2013 | 2014 | 2015 | 2016 | 2017 | 2018 | 2019 | 2020 |
| --- | --- | --- | --- | --- | --- | --- | --- | --- | --- | --- | --- |
| Total |  | 3,466  6.8 (5.6-8.1) | 3,282  7.9 (6.5-9.4) | 3,084  6.2 (5.0-7.4) | 3,038  5.7 (4.3-7.0) | 3,053  5.5 (4.4-6.7) | 3,423  6.4 (5.2-7.5) | 3,398  6.0 (4.7-7.2) | 3,470  7.5 (6.1-8.9) | 3,446  6.7 (5.3-8.0) | 3,231  6.6 (5.4-7.9) |
| Age | 19-29 | 390  10.4 (6.3-14.5) | 384  13.6 (9.3-17.9) | 389  9.1 (5.7-12.4) | 355  8.9 (4.7-13.1) | 347  6.9 (3.7-10.1) | 391  7.2 (4.6-9.8) | 380  9.7 (6.1-13.3) | 403  10.9 (7.2-14.6) | 361  10.2 (6.3-14.1) | 400  10.9 (7.1-14.7) |
|  | 30-39 | 654  8.9 (6.3-11.5) | 571  9.0 (6.0-12.1) | 552  6.9 (4.2-9.6) | 526  7.0 (4.6-9.3) | 445  6.7 (3.7-9.7) | 621  7.6 (5.0-10.3) | 499  6.8 (4.3-9.2) | 493  8.3 (4.9-11.7) | 492  7.2 (4.4-10.0) | 428  8.8 (5.6-12.1) |
|  | 40-49 | 604  4.1 (2.2-6.0) | 557  5.5 (3.1-7.8) | 594  6.2 (4.1-8.3) | 536  5.0 (3.1-7.0) | 554  4.9 (2.7-7.1) | 624  5.6 (3.6-7.6) | 626  5.7 (3.4-7.9) | 652  8.7 (6.1-11.4) | 624  6.5 (4.0-8.9) | 546  5.7 (3.2-8.1) |
|  | 50-59 | 676  5.0 (2.9-7.1) | 633  7.9 (5.1-10.8) | 598  3.7 (1.9-5.6) | 595  2.5 (1.1-3.9)* | 646  5.4 (3.2-7.5) | 641  7.1 (4.7-9.6) | 663  3.3 (1.8-4.8) | 687  5.0 (3.0-7.1) | 690  4.2 (2.4-5.9) | 579  2.0 (0.8-3.1)* |
|  | 60-69 | 563  3.8 (1.8-5.8)* | 572  1.6 (0.5-2.8)* | 469  4.0 (1.9-6.1)* | 514  2.5 (1.0-3.9)* | 532  2.8 (1.3-4.3)* | 557  4.0 (1.7-6.3)* | 620  2.8 (1.5-4.2) | 601  3.6 (1.7-5.5)* | 625  4.4 (1.8-6.9)* | 630  4.3 (2.1-6.5)* |
|  | 70+ | 579  5.1 (2.8-7.4) | 565  3.2 (1.4-4.9)* | 482  3.1 (1.4-4.7)* | 512  3.8 (1.4-6.1)* | 529  3.7 (1.6-5.8)* | 589  3.4 (1.2-5.6)* | 610  1.9 (0.8-3.0)* | 634  1.1 (0.3-2.0)* | 654  2.5 (1.3-3.6) | 648  1.7 (0.6-2.7)* |
| Number of household members | 1 | 315  - - | 356  - - | 340  - - | 355  - - | 364  - - | 424  11.9 (6.5-17.2) | 479  12.1 (6.6-17.6) | 489  17.0 (10.8-23.1) | 506  - - | 459  17.5 (12.1-22.9) |
|  | 2+ | 3,151  6.4 (5.1-7.6) | 2,926  7.4 (5.9-8.8) | 2,744  5.6 (4.5-6.7) | 2,683  5.4 (4.0-6.8) | 2,689  5.3 (4.2-6.5) | 2,999  6.0 (4.8-7.2) | 2,919  5.5 (4.2-6.7) | 2,981  6.7 (5.3-8.1) | 2,940  5.3 (4.0-6.7) | 2,772  5.9 (4.7-7.1) |
| Residential area | Urban areas | 2,779  6.7 (5.4-7.9) | 2,640  8.2 (6.6-9.8) | 2,505  6.2 (4.9-7.5) | 2,475  5.5 (4.1-6.9) | 2,485  5.9 (4.7-7.2) | 2,766  5.8 (4.6-7.0) | 2,781  6.2 (4.9-7.5) | 2,852  6.8 (5.4-8.2) | 2,779  6.9 (5.4-8.4) | 2,579  7.1 (5.7-8.5) |
|  | Rural areas | 687  8.2 (3.5-12.9)* | 642  6.6 (2.4-10.7)* | 579  5.5 (3.0-8.0) | 563  6.6 (2.2-10.9)* | 568  2.6 (0.7-4.4)* | 657  9.9 (6.4-13.4) | 617  3.9 (0.5-7.3)* | 618  13.6 (8.0-19.2) | 667  4.9 (2.2-7.7)* | 652  3.1 (1.4-4.9)* |
| Income | Lowest | 695  11.9 (8.5-15.3) | 635  11.4 (7.7-15.1) | 594  9.3 (6.4-12.3) | 591  11.2 (7.5-15.0) | 584  9.4 (6.2-12.5) | 671  8.9 (6.2-11.6) | 674  9.3 (5.9-12.6) | 694  10.7 (7.7-13.6) | 684  13.0 (9.4-16.6) | 632  12.1 (8.4-15.7) |
|  | Lower middle | 682  8.6 (5.6-11.6) | 664  10.6 (7.1-14.0) | 604  7.8 (4.9-10.7) | 603  5.9 (3.4-8.4) | 616  5.9 (3.5-8.2) | 683  8.2 (5.4-10.9) | 681  5.3 (3.0-7.5) | 696  10.6 (6.8-14.4) | 681  6.5 (3.4-9.5) | 637  9.1 (6.0-12.2) |
|  | Middle | 684  4.9 (2.8-7.1) | 639  6.5 (3.6-9.5) | 634  5.5 (3.2-7.9) | 605  4.2 (2.1-6.4)* | 613  4.7 (2.3-7.1)* | 694  5.2 (3.0-7.4) | 678  7.0 (4.6-9.4) | 693  6.9 (4.5-9.3) | 686  7.9 (5.2-10.7) | 650  5.3 (2.9-7.7) |
|  | Upper middle | 689  3.9 (1.8-5.9)* | 648  4.2 (1.8-6.6)* | 620  4.4 (2.2-6.7)* | 618  4.1 (0.2-7.9)* | 614  4.2 (2.0-6.4)* | 688  4.5 (2.2-6.7)* | 681  4.6 (2.0-7.2)* | 684  5.6 (3.2-8.0) | 696  4.5 (2.6-6.4) | 651  4.8 (2.3-7.3)* |
|  | Highest | 690  3.9 (1.8-5.9)* | 658  5.8 (2.9-8.7)* | 618  4.3 (1.9-6.7)* | 605  2.4 (0.8-4.0)* | 610  3.6 (1.6-5.6)* | 677  5.3 (3.0-7.6) | 671  3.4 (1.7-5.1)* | 693  3.2 (1.4-5.0)* | 684  1.8 (0.5-3.0)* | 647  2.4 (1.1-3.7)* |
| Education  (aged 30-59 years) | ≤High school | 1,247  9.6 (7.2-12.1) | 1,122  9.7 (7.2-12.3) | 1,100  8.6 (6.2-11.0) | 962  8.0 (5.5-10.5) | 913  9.4 (6.1-12.7) | 988  11.1 (7.9-14.2) | 868  9.0 (6.0-11.9) | 921  15.8 (11.5-20.1) | 847  13.5 (9.4-17.6) | 698  13.2 (8.9-17.5) |
|  | ≥College | 684  2.6 (1.0-4.1)* | 637  6.7 (2.9-10.4)* | 643  2.8 (1.4-4.2)* | 611  2.4 (1.3-3.5) | 632  2.5 (1.1-3.8)* | 835  2.9 (1.6-4.2) | 842  3.7 (2.2-5.2) | 851  3.2 (1.8-4.6) | 914  2.6 (1.4-3.8) | 785  2.6 (1.2-3.9)* |
| Education  (aged ≥60 years) | ≤Middle school | 990  4.0 (2.5-5.5) | 959  2.4 (1.4-3.5) | 812  4.3 (2.6-6.0) | 789  3.1 (1.8-4.5) | 784  1.6 (0.8-2.5)* | 897  4.2 (2.0-6.3)* | 931  2.3 (1.2-3.4) | 928  2.4 (1.2-3.6) | 896  3.8 (1.6-6.0)* | 799  3.5 (1.4-5.6)* |
|  | ≥ High school | 150  7.5 (0.1-14.9)** | 175  2.0 (-1.8-5.7)** | 139  0.0 (0.0-0.0) | 180  0.5 (-0.5-1.4)** | 186  4.4 (0.0-8.8)** | 203  2.4 (-0.7-5.4)** | 238  2.1 (0.0-4.2)** | 261  1.3 (0.2-2.4)* | 295  2.3 (0.6-3.9)* | 298  1.0 (-0.3-2.3)** |
| Occupation | Non-manual | 365  4.1 (1.8-6.4)* | 380  3.1 (0.6-5.6)* | 393  2.4 (0.9-3.9)* | 380  4.0 (1.8-6.1)* | 398  2.9 (1.0-4.8)* | 492  4.3 (2.2-6.5)* | 550  3.5 (1.8-5.2)* | 559  4.6 (2.1-7.1)* | 575  4.3 (2.3-6.4) | 499  4.9 (2.6-7.2) |
|  | Manual | 662  10.0 (5.9-14.1) | 598  11.2 (8.0-14.4) | 599  9.3 (5.8-12.8) | 510  8.2 (5.1-11.4) | 519  8.0 (4.3-11.7) | 565  10.7 (6.8-14.5) | 504  9.6 (5.2-14.1) | 601  14.6 (9.1-20.0) | 515  12.5 (6.8-18.1) | 424  7.7 (3.5-11.9)* |
|  | Others | 905  6.0 (4.0-7.9) | 780  7.6 (5.1-10.2) | 752  5.6 (3.5-7.7) | 684  4.2 (2.4-5.9) | 626  6.1 (3.4-8.7) | 766  7.1 (4.8-9.3) | 656  5.7 (3.5-7.8) | 612  6.9 (4.6-9.3) | 670  4.8 (2.9-6.7) | 558  6.5 (4.2-8.7) |

*coefficient of variation 25-50%

** coefficient of variation ≥50%
