# Supplementary material for: Smoking, drinking, and physical activity among Korean adults before and during the COVID-19 pandemic: a special report of the 2020 Korea National Health and Nutrition Examination Survey
Source: Epidemiol Health. 2022 Apr 25;44:e2022043. doi: 10.4178/epih.e2022043 (PMC9133597; doi:10.4178/epih.e2022043)
Supplement: Supplementary Material 4 — Numbers and age-standardized rates (%) of high-risk drinking by demographic and socioeconomic indicators among Koreans (men and women combined) aged 19 or older in the 2011-2020 Korea National Health and Nutrition Examination Survey. [file epih-44-e2022043-suppl4.docx]

Supplementary Material 4. Numbers and age-standardized rates (%) of high-risk drinking by demographic and socioeconomic indicators among Koreans (men and women combined) aged 19 or older in the 2011-2020 Korea National Health and Nutrition Examination Survey.

|  |  | 2011 | 2012 | 2013 | 2014 | 2015 | 2016 | 2017 | 2018 | 2019 | 2020 |
| --- | --- | --- | --- | --- | --- | --- | --- | --- | --- | --- | --- |
| Total |  | 6,027  14.1 (12.8-15.4) | 5,590  13.9 (12.6-15.2) | 5,341  12.6 (11.4-13.8) | 5,199  13.5 (12.1-15.0) | 5,410  13.3 (12.0-14.6) | 6,017  13.8 (12.6-15.0) | 6,116  14.2 (13.0-15.4) | 6,188  14.7 (13.4-15.9) | 6,195  12.6 (11.3-13.8) | 5,860  14.1 (12.8-15.3) |
| Age | 19-29 | 678  14.2 (10.9-17.5) | 637  14.4 (10.8-17.9) | 701  14.1 (10.7-17.6) | 594  11.9 (7.9-15.8) | 682  12.7 (9.9-15.6) | 695  13.8 (10.6-17.1) | 724  14.2 (10.7-17.7) | 762  15.9 (12.7-19.1) | 749  11.2 (8.2-14.1) | 798  11.4 (8.7-14.1) |
|  | 30-39 | 1,098  18.8 (16.0-21.7) | 960  17.2 (14.4-19.9) | 938  15.6 (12.9-18.3) | 904  17.6 (14.7-20.5) | 745  15.8 (12.4-19.2) | 1,078  16.4 (13.5-19.3) | 904  16.9 (14.3-19.5) | 909  15.2 (12.6-17.8) | 916  13.8 (11.0-16.7) | 766  16.6 (13.5-19.6) |
|  | 40-49 | 1,056  16.2 (13.7-18.8) | 958  17.2 (14.1-20.4) | 1,040  15.1 (12.8-17.3) | 901  16.4 (13.6-19.2) | 951  16.2 (13.4-19.0) | 1,124  15.8 (13.3-18.3) | 1,122  16.9 (14.5-19.4) | 1,129  18.1 (15.5-20.7) | 1,107  15.3 (12.9-17.8) | 965  19.0 (15.7-22.3) |
|  | 50-59 | 1,181  14.1 (11.6-16.6) | 1,067  13.3 (10.1-16.5) | 1,013  12.1 (9.6-14.7) | 996  14.5 (11.9-17.1) | 1,130  13.8 (11.3-16.4) | 1,092  15.4 (13.0-17.9) | 1,205  14.5 (12.3-16.7) | 1,187  14.6 (12.2-17.1) | 1,166  13.9 (11.8-16.1) | 1,060  13.9 (11.6-16.2) |
|  | 60-69 | 1,027  6.5 (4.7-8.3) | 1,004  6.5 (4.4-8.6) | 850  4.6 (3.0-6.2) | 919  7.4 (5.1-9.6) | 992  9.1 (7.2-11.1) | 1,000  9.0 (6.8-11.1) | 1,090  9.1 (7.1-11.1) | 1,101  11.2 (8.8-13.7) | 1,113  10.4 (8.4-12.4) | 1,113  10.9 (9.0-12.9) |
|  | 70+ | 987  2.0 (0.9-3.2)* | 964  2.8 (1.5-4.0) | 799  1.7 (0.8-2.6)* | 885  3.3 (2.0-4.5) | 910  2.5 (1.2-3.8)* | 1,028  2.7 (1.6-3.8) | 1,071  3.2 (2.2-4.3) | 1,100  3.0 (1.9-4.2) | 1,144  4.7 (3.3-6.2) | 1,158  3.5 (2.3-4.6) |
| Number of household members | 1 | 448  23.9 (15.3-32.5) | 486  21.6 (13.4-29.7) | 497  18.3 (11.7-25.0) | 519  21.2 (13.8-28.6) | 568  16.3 (10.6-21.9) | 677  16.5 (12.4-20.5) | 806  16.6 (12.7-20.5) | 802  15.6 (11.1-20.0) | 825  20.2 (14.7-25.7) | 803  18.0 (13.9-22.1) |
|  | 2+ | 5,579  13.7 (12.4-15.0) | 5,104  13.5 (12.2-14.9) | 4,843  12.3 (11.1-13.5) | 4,680  13.1 (11.7-14.5) | 4,842  13.1 (11.8-14.4) | 5,340  13.7 (12.4-14.9) | 5,310  14.0 (12.7-15.3) | 5,386  14.6 (13.2-15.9) | 5,370  12.0 (10.7-13.2) | 5,057  13.7 (12.3-15.1) |
| Residential area | Urban areas | 4,813  13.6 (12.2-14.9) | 4,465  13.9 (12.5-15.3) | 4,311  12.4 (11.1-13.7) | 4,197  13.2 (11.7-14.7) | 4,368  13.1 (11.7-14.6) | 4,854  13.3 (12.0-14.6) | 4,989  14.3 (13.0-15.6) | 5,063  14.3 (13.0-15.6) | 4,962  12.1 (10.7-13.5) | 4,662  13.9 (12.5-15.3) |
|  | Rural areas | 1,214  16.5 (12.3-20.7) | 1,125  14.2 (10.6-17.8) | 1,030  13.0 (10.0-16.0) | 1,002  16.1 (11.2-20.9) | 1,042  13.9 (11.0-16.8) | 1,163  17.1 (13.0-21.2) | 1,127  13.0 (9.5-16.6) | 1,125  16.3 (11.7-20.8) | 1,233  15.4 (12.5-18.3) | 1,198  14.8 (11.6-18.0) |
| Income | Lowest | 1,203  15.8 (13.1-18.5) | 1,079  13.4 (10.7-16.1) | 1,036  11.3 (9.0-13.6) | 1,010  14.5 (11.7-17.3) | 1,046  14.4 (11.5-17.3) | 1,193  13.7 (11.2-16.1) | 1,211  14.3 (11.8-16.8) | 1,236  14.2 (11.7-16.7) | 1,238  14.2 (11.7-16.6) | 1,150  13.2 (10.7-15.7) |
|  | Lower middle | 1,185  14.9 (11.9-17.9) | 1,116  12.4 (9.4-15.4) | 1,053  11.7 (9.1-14.2) | 1,039  14.1 (11.3-17.0) | 1,078  13.7 (11.2-16.2) | 1,202  14.6 (12.1-17.2) | 1,216  15.5 (13.0-18.0) | 1,236  15.9 (13.4-18.4) | 1,222  14.9 (12.2-17.6) | 1,162  14.7 (12.1-17.2) |
|  | Middle | 1,191  12.0 (9.0-14.9) | 1,087  16.5 (13.0-19.9) | 1,080  13.6 (10.9-16.2) | 1,037  12.8 (9.8-15.7) | 1,082  12.0 (9.2-14.8) | 1,202  10.7 (8.3-13.1) | 1,222  14.9 (11.8-17.9) | 1,243  14.4 (11.0-17.9) | 1,225  12.2 (9.5-15.0) | 1,173  14.9 (12.0-17.8) |
|  | Upper middle | 1,192  13.1 (10.5-15.6) | 1,109  12.5 (10.3-14.7) | 1,064  13.9 (10.9-16.8) | 1,054  12.4 (9.6-15.1) | 1,084  14.1 (11.0-17.3) | 1,203  15.6 (12.7-18.6) | 1,228  13.1 (10.3-15.8) | 1,222  16.4 (13.2-19.6) | 1,244  10.8 (8.6-13.0) | 1,176  13.0 (10.1-15.9) |
|  | Highest | 1,208  14.6 (11.6-17.7) | 1,134  15.5 (12.5-18.6) | 1,080  12.5 (9.7-15.3) | 1,040  13.2 (9.7-16.7) | 1,089  12.4 (9.5-15.3) | 1,198  14.4 (11.7-17.0) | 1,220  13.2 (10.5-15.9) | 1,231  12.3 (9.7-14.8) | 1,239  11.1 (8.5-13.8) | 1,179  14.7 (12.1-17.4) |
| Education  (aged 30-59 years) | ≤High school | 1,977  17.1 (14.5-19.7) | 1,751  18.3 (15.7-20.8) | 1,742  15.8 (13.5-18.1) | 1,500  18.8 (16.4-21.1) | 1,453  18.6 (15.5-21.8) | 1,553  16.9 (14.5-19.3) | 1,451  21.6 (18.6-24.5) | 1,484  20.7 (17.9-23.6) | 1,380  17.5 (14.6-20.4) | 1,184  21.7 (18.4-24.9) |
|  | ≥College | 1,347  16.2 (13.7-18.8) | 1,230  14.9 (12.3-17.4) | 1,247  14.5 (12.0-16.9) | 1,157  15.2 (12.7-17.8) | 1,164  12.1 (9.5-14.7) | 1,611  15.5 (13.4-17.6) | 1,609  13.1 (11.2-15.0) | 1,619  13.7 (11.7-15.7) | 1,706  12.5 (10.6-14.5) | 1,477  13.8 (11.6-15.9) |
| Education  (aged ≥60 years) | ≤Middle school | 1,507  4.5 (3.2-5.8) | 1,436  4.7 (3.2-6.3) | 1,232  3.2 (2.0-4.4) | 1,203  5.4 (3.6-7.2) | 1,210  6.5 (4.6-8.4) | 1,376  5.2 (3.6-6.9) | 1,410  6.3 (4.7-7.9) | 1,406  8.1 (6.0-10.1) | 1,368  7.1 (5.3-8.9) | 1,218  5.5 (4.0-6.9) |
|  | ≥ High school | 505  4.9 (2.9-6.9) | 527  5.7 (2.9-8.6)* | 417  3.3 (1.6-5.0)* | 497  5.5 (3.3-7.6) | 539  6.3 (3.9-8.8) | 574  8.0 (5.5-10.5) | 634  7.4 (5.0-9.7) | 699  7.6 (5.4-9.8) | 734  9.2 (6.9-11.6) | 746  8.7 (6.5-10.8) |
| Occupation | Non-manual | 915  19.6 (16.4-22.8) | 885  18.0 (14.4-21.7) | 861  15.7 (12.9-18.5) | 836  18.2 (15.0-21.4) | 826  15.2 (12.3-18.2) | 1,071  17.5 (14.7-20.3) | 1,151  15.0 (12.9-17.0) | 1,143  15.4 (13.2-17.7) | 1,162  13.1 (10.3-15.8) | 1,006  14.7 (12.0-17.3) |
|  | Manual | 1,398  21.5 (18.5-24.5) | 1,207  19.6 (16.5-22.6) | 1,262  20.1 (17.4-22.8) | 1,059  23.4 (20.2-26.6) | 1,052  20.6 (17.0-24.2) | 1,204  20.1 (17.4-22.9) | 1,130  24.2 (20.6-27.9) | 1,240  20.1 (17.2-23.1) | 1,124  20.8 (17.5-24.1) | 964  23.4 (19.4-27.3) |
|  | Others | 1,011  6.6 (4.4-8.8) | 887  9.3 (6.2-12.3) | 868  5.9 (4.0-7.8) | 763  6.8 (4.8-8.8) | 735  6.0 (4.1-7.9) | 890  9.0 (6.6-11.5) | 781  7.9 (5.4-10.3) | 716  10.2 (7.8-12.7) | 794  6.8 (4.6-9.0) | 688  10.4 (7.5-13.3) |

*coefficient of variation 25-50%
