# Supplementary material for: Smoking, drinking, and physical activity among Korean adults before and during the COVID-19 pandemic: a special report of the 2020 Korea National Health and Nutrition Examination Survey
Source: Epidemiol Health. 2022 Apr 25;44:e2022043. doi: 10.4178/epih.e2022043 (PMC9133597; doi:10.4178/epih.e2022043)
Supplement: Supplementary Material 5 — Numbers and age-standardized rates (%) of high-risk drinking by demographic and socioeconomic indicators among Korean men aged 19 or older in the 2011-2020 Korea National Health and Nutrition Examination Survey. [file epih-44-e2022043-suppl5.docx]

Supplementary Material 5. Numbers and age-standardized rates (%) of high-risk drinking by demographic and socioeconomic indicators among Korean men aged 19 or older in the 2011-2020 Korea National Health and Nutrition Examination Survey.

|  |  | 2011 | 2012 | 2013 | 2014 | 2015 | 2016 | 2017 | 2018 | 2019 | 2020 |
| --- | --- | --- | --- | --- | --- | --- | --- | --- | --- | --- | --- |
| Total |  | 2,559  23.2 (20.9-25.5) | 2,308  21.9 (19.5-24.3) | 2,255  19.7 (17.7-21.7) | 2,155  20.7 (18.2-23.2) | 2,354  20.8 (18.5-23.1) | 2,593  21.2 (19.1-23.3) | 2,719  21.0 (19.0-23.0) | 2,717  20.8 (19.0-22.7) | 2,746  18.6 (16.6-20.6) | 2,629  21.6 (19.6-23.6) |
| Age | 19-29 | 288  19.7 (14.3-25.1) | 253  19.3 (13.9-24.6) | 312  18.7 (13.1-24.3) | 239  14.6 (8.9-20.3) | 335  14.6 (10.0-19.2) | 304  17.7 (12.5-22.8) | 344  17.0 (12.2-21.7) | 359  16.7 (12.2-21.2) | 388  13.1 (8.7-17.4) | 398  14.3 (10.0-18.5) |
|  | 30-39 | 444  30.5 (25.4-35.6) | 389  25.4 (20.8-30.1) | 386  23.7 (19.3-28.0) | 378  26.2 (20.8-31.5) | 299  25.1 (19.6-30.7) | 457  23.5 (18.6-28.5) | 405  24.1 (19.6-28.6) | 416  20.6 (16.8-24.3) | 424  19.0 (14.5-23.4) | 338  23.3 (18.5-28.1) |
|  | 40-49 | 452  28.1 (23.1-33.1) | 401  27.8 (22.3-33.3) | 446  25.9 (21.8-30.1) | 365  26.0 (21.1-30.8) | 397  27.6 (22.5-32.8) | 500  25.7 (21.5-29.8) | 496  27.2 (22.9-31.4) | 477  27.7 (23.2-32.1) | 483  23.2 (19.4-27.0) | 419  30.1 (24.8-35.4) |
|  | 50-59 | 505  25.8 (21.1-30.5) | 434  24.5 (18.9-30.1) | 415  20.3 (15.7-24.9) | 400  24.8 (20.1-29.5) | 484  23.1 (18.6-27.5) | 451  26.0 (21.5-30.4) | 541  23.5 (19.7-27.4) | 500  24.1 (19.9-28.3) | 477  24.0 (19.8-28.3) | 481  24.3 (20.2-28.5) |
|  | 60-69 | 462  12.7 (9.2-16.3) | 432  13.1 (9.0-17.2) | 379  8.9 (5.9-11.9) | 406  14.0 (9.6-18.4) | 460  16.7 (13.2-20.3) | 442  17.5 (13.2-21.8) | 470  15.2 (11.4-18.9) | 500  20.6 (16.0-25.1) | 488  17.7 (14.3-21.1) | 483  21.2 (17.3-25.2) |
|  | 70+ | 408  4.4 (1.8-7.1)* | 399  6.8 (3.7-9.9) | 317  4.3 (2.0-6.6)* | 367  6.6 (3.8-9.4) | 379  5.8 (2.6-8.9)* | 439  6.3 (3.5-9.1) | 463  7.2 (4.8-9.7) | 465  7.2 (4.3-10.0) | 486  10.5 (7.3-13.8) | 510  7.9 (5.3-10.6) |
| Number of household members | 1 | 133  - - | 130  - - | 157  - - | 163  - - | 203  19.7 (11.9-27.4) | 252  23.1 (17.0-29.2) | 329  19.4 (14.3-24.5) | 311  20.6 (14.5-26.8) | 318  24.9 (18.1-31.6) | 344  20.0 (15.4-24.6) |
|  | 2+ | 2,426  22.9 (20.6-25.3) | 2,178  21.6 (19.2-24.0) | 2,097  19.5 (17.5-21.6) | 1,992  20.2 (17.7-22.7) | 2,151  20.8 (18.5-23.1) | 2,341  21.1 (18.9-23.4) | 2,390  21.3 (19.0-23.5) | 2,406  20.7 (18.7-22.7) | 2,428  18.0 (15.9-20.1) | 2,285  21.9 (19.7-24.1) |
| Residential area | Urban areas | 2,032  22.0 (19.6-24.5) | 1,825  21.8 (19.2-24.4) | 1,804  19.8 (17.6-21.9) | 1,718  20.6 (17.9-23.2) | 1,880  20.7 (18.2-23.3) | 2,088  19.9 (17.8-22.1) | 2,208  21.6 (19.4-23.7) | 2,211  20.5 (18.5-22.6) | 2,180  17.9 (15.7-20.1) | 2,083  21.3 (19.2-23.4) |
|  | Rural areas | 527  28.4 (21.2-35.6) | 483  21.8 (15.4-28.3) | 451  18.4 (13.7-23.2) | 437  21.7 (14.9-28.5) | 474  20.4 (15.3-25.5) | 505  29.3 (22.4-36.2) | 511  16.7 (11.8-21.5) | 506  20.9 (17.5-24.3) | 566  22.4 (18.4-26.4) | 546  23.5 (18.5-28.4) |
| Income | Lowest | 508  24.6 (19.9-29.2) | 444  19.9 (15.3-24.6) | 442  17.5 (13.7-21.4) | 419  21.0 (16.2-25.7) | 461  21.4 (16.6-26.2) | 522  20.8 (16.7-24.9) | 537  18.5 (14.8-22.2) | 540  20.9 (16.7-25.1) | 553  17.8 (14.2-21.3) | 518  17.9 (14.1-21.7) |
|  | Lower middle | 503  26.2 (20.9-31.6) | 452  19.8 (14.8-24.9) | 448  16.3 (12.5-20.1) | 435  19.8 (15.0-24.7) | 460  21.9 (17.8-26.1) | 518  21.0 (17.0-24.9) | 535  23.1 (18.8-27.3) | 541  21.2 (17.3-25.0) | 542  23.5 (19.0-27.9) | 525  20.7 (16.5-24.9) |
|  | Middle | 506  20.7 (15.3-26.2) | 448  25.7 (19.6-31.8) | 446  22.2 (17.9-26.5) | 431  19.7 (15.0-24.4) | 469  16.5 (12.1-20.9) | 507  16.3 (12.3-20.3) | 544  21.9 (17.3-26.5) | 550  18.9 (14.2-23.7) | 539  16.9 (13.0-20.8) | 523  23.8 (19.0-28.6) |
|  | Upper middle | 503  20.0 (15.3-24.7) | 461  19.2 (15.3-23.2) | 444  23.3 (18.3-28.3) | 433  19.8 (15.0-24.5) | 470  25.0 (19.5-30.6) | 515  26.1 (21.3-30.9) | 547  20.3 (16.2-24.4) | 538  23.9 (19.3-28.6) | 548  17.5 (13.6-21.5) | 525  20.3 (15.3-25.3) |
|  | Highest | 517  24.4 (19.4-29.3) | 476  25.8 (21.1-30.5) | 461  20.4 (15.5-25.3) | 434  22.9 (16.8-29.0) | 479  20.3 (15.7-24.9) | 522  22.2 (17.7-26.7) | 549  21.2 (16.6-25.8) | 538  19.1 (14.7-23.5) | 552  17.8 (13.3-22.2) | 532  25.6 (21.1-30.1) |
| Education  (aged 30-59 years) | ≤High school | 730  30.1 (25.4-34.9) | 629  30.3 (25.6-35.0) | 642  26.0 (21.9-30.0) | 537  29.3 (24.7-33.8) | 540  32.1 (26.5-37.6) | 565  25.9 (21.6-30.2) | 583  31.0 (25.4-36.5) | 563  31.0 (26.3-35.6) | 533  23.2 (18.9-27.5) | 486  32.5 (27.4-37.7) |
|  | ≥College | 663  25.3 (21.3-29.2) | 593  22.6 (18.9-26.3) | 604  22.4 (18.8-26.0) | 546  23.8 (19.8-27.8) | 532  19.6 (15.5-23.8) | 776  23.6 (20.1-27.2) | 767  21.1 (17.8-24.4) | 768  20.0 (17.0-23.0) | 792  19.7 (16.3-23.1) | 692  22.0 (18.4-25.6) |
| Education  (aged ≥60 years) | ≤Middle school | 515  10.9 (7.5-14.2) | 477  12.0 (8.1-16.0) | 418  8.0 (5.3-10.7) | 410  12.2 (8.1-16.4) | 424  14.5 (10.4-18.6) | 478  12.9 (8.6-17.2) | 480  13.0 (9.0-17.0) | 476  18.7 (13.5-23.8) | 472  14.9 (11.4-18.4) | 419  14.0 (10.2-17.7) |
|  | ≥ High school | 355  6.6 (3.8-9.4) | 352  8.1 (4.1-12.2)* | 278  5.0 (2.5-7.4)* | 317  8.5 (5.1-11.9) | 353  9.4 (5.8-13.1) | 371  12.1 (8.4-15.9) | 396  10.7 (7.2-14.2) | 438  12.0 (8.6-15.4) | 439  13.9 (10.5-17.4) | 448  14.3 (11.0-17.6) |
| Occupation | Non-manual | 550  27.5 (23.0-31.9) | 505  25.6 (21.0-30.3) | 468  23.1 (18.9-27.2) | 456  25.4 (20.8-30.0) | 428  23.3 (18.8-27.7) | 579  25.1 (20.8-29.4) | 601  22.3 (18.6-25.9) | 584  21.4 (18.2-24.6) | 587  19.1 (14.7-23.5) | 507  21.1 (17.2-25.0) |
|  | Manual | 736  29.9 (25.8-33.9) | 609  26.8 (22.4-31.3) | 663  25.6 (22.1-29.1) | 548  28.7 (24.3-33.2) | 533  28.4 (23.5-33.3) | 639  25.2 (21.3-29.1) | 626  29.3 (24.5-34.1) | 639  26.7 (22.6-30.7) | 609  25.2 (20.9-29.5) | 540  31.8 (26.6-36.9) |
|  | Others | 106  - - | 107  21.8 (9.7-33.8)* | 116  17.4 (9.5-25.4) | 79  - - | 109  - - | 124  24.0 (15.3-32.6) | 124  17.0 (8.8-25.3) | 104  21.0 (12.9-29.0) | 124  13.3 (6.0-20.5)* | 130  21.1 (11.9-30.3) |

*coefficient of variation 25-50%
