# Supplementary material for: Smoking, drinking, and physical activity among Korean adults before and during the COVID-19 pandemic: a special report of the 2020 Korea National Health and Nutrition Examination Survey
Source: Epidemiol Health. 2022 Apr 25;44:e2022043. doi: 10.4178/epih.e2022043 (PMC9133597; doi:10.4178/epih.e2022043)
Supplement: Supplementary Material 6 — Numbers and age-standardized rates (%) of high-risk drinking by demographic and socioeconomic indicators among Korean women aged 19 or older in the 2011-2020 Korea National Health and Nutrition Examination Survey. [file epih-44-e2022043-suppl6.docx]

Supplementary Material 6. Numbers and age-standardized rates (%) of high-risk drinking by demographic and socioeconomic indicators among Korean women aged 19 or older in the 2011-2020 Korea National Health and Nutrition Examination Survey.

|  |  | 2011 | 2012 | 2013 | 2014 | 2015 | 2016 | 2017 | 2018 | 2019 | 2020 |
| --- | --- | --- | --- | --- | --- | --- | --- | --- | --- | --- | --- |
| Total |  | 3,468  4.9 (3.9-6.0) | 3,282  6.0 (4.6-7.3) | 3,086  5.5 (4.4-6.5) | 3,044  6.6 (5.3-7.9) | 3,056  5.8 (4.7-6.9) | 3,424  6.3 (5.2-7.3) | 3,397  7.2 (5.8-8.6) | 3,471  8.4 (7.0-9.8) | 3,449  6.5 (5.4-7.7) | 3,231  6.3 (5.1-7.4) |
| Age | 19-29 | 390  8.2 (4.9-11.6) | 384  9.2 (4.5-13.9)* | 389  8.8 (5.6-11.9) | 355  8.9 (5.4-12.4) | 347  10.6 (7.0-14.2) | 391  9.6 (6.4-12.7) | 380  11.1 (6.7-15.5) | 403  14.9 (10.7-19.2) | 361  9.0 (5.9-12.1) | 400  8.3 (5.0-11.7) |
|  | 30-39 | 654  6.9 (4.7-9.1) | 571  8.4 (5.3-11.6) | 552  7.6 (5.1-10.1) | 526  9.1 (5.8-12.4) | 446  6.2 (4.0-8.4) | 621  8.6 (6.2-11.1) | 499  9.0 (6.4-11.7) | 493  9.5 (6.5-12.4) | 492  8.1 (5.5-10.8) | 428  9.1 (6.1-12.1) |
|  | 40-49 | 604  4.2 (2.4-5.9) | 557  6.4 (4.2-8.7) | 594  4.4 (2.6-6.3) | 536  7.1 (4.9-9.4) | 554  4.7 (2.7-6.7) | 624  5.7 (3.5-7.8) | 626  6.4 (4.3-8.5) | 652  8.0 (5.6-10.5) | 624  7.2 (4.7-9.8) | 546  7.3 (5.0-9.6) |
|  | 50-59 | 676  2.6 (1.0-4.3)* | 633  2.5 (0.8-4.2)* | 598  4.2 (2.1-6.3)* | 596  4.5 (2.5-6.6) | 646  4.7 (2.6-6.9) | 641  4.9 (3.0-6.8) | 664  5.5 (3.5-7.4) | 687  5.1 (3.1-7.2) | 689  4.0 (2.5-5.5) | 579  3.5 (2.0-4.9) |
|  | 60-69 | 565  0.8 (-0.1-1.7)** | 572  0.7 (0.0-1.5)* | 471  0.8 (0.0-1.7)** | 513  1.4 (0.3-2.5)* | 532  2.0 (0.3-3.7)* | 558  0.9 (0.1-1.8)* | 620  3.4 (1.1-5.6)* | 601  2.3 (1.2-3.5)* | 625  3.5 (1.7-5.3)* | 630  1.0 (0.3-1.8)* |
|  | 70+ | 579  0.5 (-0.2-1.2)** | 565  0.2 (-0.2-0.7)** | 482  0.0 (0.0-0.0) | 518  1.0 (0.0-2.0)** | 531  0.3 (-0.2-0.9)** | 589  0.3 (-0.1-0.7)** | 608  0.5 (0.0-1.0)* | 635  0.2 (-0.1-0.6)** | 658  0.8 (0.0-1.5)* | 648  0.3 (-0.1-0.7)** |
| Number of household members | 1 | 315  - - | 356  - - | 340  - - | 356  - - | 365  - - | 425  8.6 (4.3-12.9)* | 477  10.9 (5.6-16.1) | 491  8.5 (4.1-12.9)* | 507  - - | 459  14.8 (8.8-20.7) |
|  | 2+ | 3,153  4.7 (3.7-5.7) | 2,926  5.5 (4.1-6.9) | 2,746  5.3 (4.2-6.4) | 2,688  6.4 (5.1-7.7) | 2,691  5.6 (4.5-6.7) | 2,999  6.1 (5.0-7.2) | 2,920  6.9 (5.5-8.3) | 2,980  8.4 (6.9-9.9) | 2,942  5.9 (4.7-7.0) | 2,772  5.7 (4.6-6.8) |
| Residential area | Urban areas | 2,781  5.1 (4.0-6.2) | 2,640  6.3 (4.7-7.8) | 2,507  5.3 (4.1-6.5) | 2,479  6.2 (4.9-7.4) | 2,488  5.8 (4.6-7.0) | 2,766  6.6 (5.4-7.7) | 2,781  7.0 (5.6-8.4) | 2,852  8.1 (6.7-9.5) | 2,782  6.4 (5.1-7.6) | 2,579  6.4 (5.2-7.7) |
|  | Rural areas | 687  4.5 (0.6-8.4)* | 642  3.9 (1.2-6.7)* | 579  6.7 (4.2-9.3) | 565  9.8 (3.8-15.8)* | 568  5.9 (2.7-9.2)* | 658  4.6 (2.2-7.0)* | 616  8.8 (3.8-13.9)* | 619  11.7 (4.8-18.6)* | 667  7.7 (4.0-11.3) | 652  5.1 (2.5-7.7)* |
| Income | Lowest | 695  7.0 (4.0-10.1) | 635  7.0 (4.2-9.8) | 594  5.1 (3.0-7.3) | 591  8.7 (5.6-11.9) | 585  7.3 (4.5-10.0) | 671  6.0 (3.7-8.3) | 674  10.5 (7.1-13.9) | 696  7.7 (5.0-10.5) | 685  10.6 (7.2-14.1) | 632  8.4 (5.1-11.7) |
|  | Lower middle | 682  3.7 (1.7-5.6)* | 664  5.6 (2.8-8.5)* | 605  6.9 (4.2-9.6) | 604  8.2 (5.4-11.1) | 618  6.4 (3.7-9.1) | 684  8.5 (5.8-11.2) | 681  7.3 (4.6-10.0) | 695  11.0 (7.6-14.4) | 680  6.2 (3.9-8.5) | 637  8.4 (5.7-11.1) |
|  | Middle | 685  3.1 (1.7-4.5) | 639  7.1 (3.9-10.4) | 634  5.5 (3.2-7.8) | 606  5.9 (3.4-8.3) | 613  7.5 (4.6-10.4) | 695  5.2 (3.1-7.3) | 678  7.3 (4.7-10.0) | 693  9.7 (6.3-13.2) | 686  7.7 (4.8-10.5) | 650  6.2 (3.7-8.8) |
|  | Upper middle | 689  6.2 (3.6-8.9) | 648  5.5 (3.0-8.0) | 620  5.1 (2.6-7.5) | 621  5.3 (3.0-7.6) | 614  3.6 (1.6-5.5)* | 688  5.4 (3.1-7.8) | 681  5.7 (2.8-8.6)* | 684  8.3 (5.2-11.4) | 696  4.1 (2.1-6.1) | 651  5.5 (3.0-7.9) |
|  | Highest | 691  4.4 (2.4-6.4) | 658  4.8 (1.6-7.9)* | 619  4.8 (2.5-7.1) | 606  4.0 (2.1-6.0) | 610  4.6 (2.4-6.7) | 676  5.8 (3.3-8.4) | 671  5.2 (3.0-7.4) | 693  5.4 (3.0-7.8) | 687  4.6 (2.3-7.0)* | 647  3.2 (1.4-4.9)* |
| Education  (aged 30-59 years) | ≤High school | 1,247  6.1 (4.0-8.2) | 1,122  8.4 (6.1-10.7) | 1,100  7.4 (5.3-9.5) | 963  10.2 (7.2-13.1) | 913  7.7 (5.1-10.3) | 988  9.9 (7.1-12.6) | 868  13.2 (9.9-16.4) | 921  11.9 (8.6-15.1) | 847  12.7 (8.9-16.5) | 698  11.1 (7.5-14.6) |
|  | ≥College | 684  3.7 (2.0-5.4) | 637  2.8 (1.4-4.1) | 643  3.5 (2.0-5.0) | 611  3.9 (2.1-5.7) | 632  2.6 (1.2-4.0)* | 835  4.2 (2.7-5.6) | 842  3.5 (2.3-4.7) | 851  5.6 (3.7-7.4) | 914  4.0 (2.7-5.3) | 785  4.3 (2.9-5.7) |
| Education  (aged ≥60 years) | ≤Middle school | 992  0.7 (0.0-1.3)** | 959  0.5 (0.0-1.1)* | 814  0.6 (0.0-1.2)** | 793  1.5 (0.5-2.5)* | 786  1.8 (0.3-3.2)* | 898  0.8 (0.2-1.5)* | 930  2.4 (1.1-3.8)* | 930  2.0 (1.0-3.0)* | 896  2.7 (1.1-4.2)* | 799  0.6 (0.1-1.1)* |
|  | ≥ High school | 150  0.6 (-0.6-1.8)** | 175  0.4 (-0.4-1.1)** | 139  0.0 (0.0-0.0) | 180  0.4 (-0.4-1.3)** | 186  0.4 (-0.4-1.2)** | 203  0.2 (-0.2-0.7)** | 238  1.6 (-0.4-3.6)** | 261  0.4 (-0.4-1.1)** | 295  1.8 (0.4-3.2)* | 298  0.4 (-0.2-1.0)** |
| Occupation | Non-manual | 365  3.8 (1.5-6.2)* | 380  3.3 (1.2-5.4)* | 393  3.4 (1.5-5.2)* | 380  4.8 (2.3-7.3)* | 398  3.2 (1.3-5.0)* | 492  4.8 (2.6-7.0) | 550  4.3 (2.6-6.0) | 559  6.6 (4.0-9.3) | 575  4.6 (2.6-6.5) | 499  6.1 (3.8-8.3) |
|  | Manual | 662  8.7 (5.1-12.3) | 598  8.5 (5.0-12.0) | 599  11.6 (7.8-15.3) | 511  14.5 (9.4-19.5) | 519  7.5 (3.7-11.2)* | 565  12.2 (8.1-16.3) | 504  15.9 (10.1-21.8) | 601  9.9 (5.9-13.9) | 515  13.6 (9.1-18.2) | 424  7.8 (4.0-11.6) |
|  | Others | 905  4.2 (2.8-5.7) | 780  6.6 (4.2-9.0) | 752  3.4 (1.9-5.0) | 684  4.6 (2.9-6.2) | 626  4.9 (3.0-6.7) | 766  5.8 (3.8-7.7) | 657  6.0 (3.8-8.3) | 612  8.0 (5.5-10.6) | 670  5.5 (3.2-7.8) | 558  7.3 (4.6-10.0) |

*coefficient of variation 25-50%

** coefficient of variation ≥50%
