# Supplementary material for: Smoking, drinking, and physical activity among Korean adults before and during the COVID-19 pandemic: a special report of the 2020 Korea National Health and Nutrition Examination Survey
Source: Epidemiol Health. 2022 Apr 25;44:e2022043. doi: 10.4178/epih.e2022043 (PMC9133597; doi:10.4178/epih.e2022043)
Supplement: Supplementary Material 7 — Numbers and age-standardized rates (%) of inadequate physical activity by demographic and socioeconomic indicators among Koreans (men and women combined) aged 19 or older in the 2014-2020 Korea National Health and Nutrition Examination Survey. [file epih-44-e2022043-suppl7.docx]

Supplementary Material 7. Numbers and age-standardized rates (%) of inadequate physical activity by demographic and socioeconomic indicators among Koreans (men and women combined) aged 19 or older in the 2014-2020 Korea National Health and Nutrition Examination Survey.

|  |  | 2014 | 2015 | 2016 | 2017 | 2018 | 2019 | 2020 |
| --- | --- | --- | --- | --- | --- | --- | --- | --- |
| Total |  | 4,973  41.7 (39.9-43.5) | 5,065  47.3 (45.4-49.2) | 5,801  50.6 (48.6-52.6) | 5,810  51.5 (49.6-53.3) | 5,962  52.4 (50.4-54.4) | 5,918  52.2 (50.3-54.1) | 5,398  54.4 (52.6-56.2) |
| Age | 19-29 | 581  27.7 (23.8-31.7) | 645  33.3 (29.0-37.6) | 667  38.1 (33.6-42.6) | 704  34.4 (30.4-38.4) | 748  36.2 (31.3-41.0) | 723  37.9 (33.8-42.1) | 778  42.2 (38.3-46.0) |
|  | 30-39 | 862  42.0 (38.2-45.8) | 695  48.8 (44.0-53.5) | 1,036  50.9 (47.2-54.5) | 857  52.5 (48.2-56.7) | 881  49.3 (44.9-53.6) | 882  50.8 (46.6-55.0) | 733  52.9 (48.4-57.3) |
|  | 40-49 | 850  42.8 (39.1-46.5) | 884  45.5 (41.6-49.3) | 1,081  52.1 (48.4-55.8) | 1,061  52.5 (48.7-56.3) | 1,081  54.2 (50.6-57.7) | 1,076  54.7 (51.6-57.8) | 916  57.2 (54.2-60.2) |
|  | 50-59 | 959  43.7 (40.1-47.3) | 1,051  53.8 (49.9-57.7) | 1,052  54.6 (50.9-58.3) | 1,148  55.6 (52.1-59.0) | 1,140  62.1 (58.7-65.6) | 1,131  58.6 (55.1-62.0) | 1,012  60.1 (56.0-64.2) |
|  | 60-69 | 891  52.2 (48.0-56.4) | 939  54.3 (50.3-58.2) | 971  54.2 (50.6-57.8) | 1,038  64.2 (60.5-68.0) | 1,065  61.3 (57.5-65.2) | 1,071  60.7 (57.1-64.2) | 1,048  59.5 (56.2-62.8) |
|  | 70+ | 830  62.9 (59.2-66.5) | 851  70.2 (66.3-74.2) | 994  71.5 (68.1-74.8) | 1,002  72.8 (69.4-76.3) | 1,047  76.6 (73.4-79.9) | 1,035  69.6 (66.0-73.3) | 911  70.6 (67.1-74.2) |
| Number of household members | 1 | 486  40.6 (33.8-47.4) | 543  40.7 (33.5-47.9) | 650  49.6 (43.7-55.4) | 758  50.6 (45.4-55.8) | 772  48.7 (42.9-54.6) | 778  52.0 (46.2-57.9) | 705  51.7 (45.5-57.8) |
|  | 2+ | 4,487  41.8 (39.9-43.6) | 4,522  47.6 (45.6-49.6) | 5,151  50.7 (48.7-52.7) | 5,052  51.4 (49.5-53.4) | 5,190  52.8 (50.7-54.8) | 5,140  52.2 (50.2-54.3) | 4,693  54.6 (52.6-56.5) |
| Residential area | Urban areas | 4,043  39.9 (38.1-41.7) | 4,117  45.6 (43.6-47.6) | 4,685  49.2 (47.1-51.3) | 4,763  50.4 (48.4-52.4) | 4,902  51.2 (49.0-53.4) | 4,754  50.7 (48.7-52.6) | 4,320  52.8 (50.8-54.8) |
|  | Rural areas | 930  50.8 (44.4-57.1) | 948  55.5 (48.5-62.5) | 1,116  58.4 (52.5-64.3) | 1,047  57.2 (52.5-61.9) | 1,060  58.9 (53.8-64.0) | 1,164  60.6 (53.8-67.5) | 1,078  64.1 (60.1-68.0) |
| Income | Lowest | 962  44.2 (40.4-48.1) | 973  47.6 (43.2-52.1) | 1,145  57.4 (53.6-61.3) | 1,138  52.8 (49.3-56.2) | 1,174  55.4 (51.7-59.1) | 1,154  55.4 (51.7-59.0) | 1,028  58.4 (54.1-62.7) |
|  | Lower middle | 996  41.4 (38.0-44.8) | 1,011  47.9 (43.5-52.3) | 1,161  52.9 (49.4-56.4) | 1,150  52.9 (48.7-57.0) | 1,190  53.9 (50.1-57.6) | 1,169  53.2 (49.1-57.2) | 1,074  54.9 (50.7-59.0) |
|  | Middle | 1,000  42.6 (39.1-46.2) | 1,013  48.9 (44.7-53.1) | 1,159  51.4 (48.1-54.7) | 1,164  52.1 (48.2-55.9) | 1,194  54.5 (50.6-58.4) | 1,171  52.5 (48.6-56.4) | 1,099  54.0 (50.3-57.8) |
|  | Upper middle | 1,007  39.7 (36.1-43.3) | 1,029  50.0 (46.1-53.9) | 1,156  46.2 (42.5-50.0) | 1,168  51.4 (47.6-55.2) | 1,188  49.9 (46.1-53.7) | 1,200  49.1 (45.5-52.8) | 1,091  53.3 (49.4-57.1) |
|  | Highest | 992  40.6 (36.3-44.8) | 1,017  42.3 (38.3-46.3) | 1,166  45.0 (41.4-48.5) | 1,174  48.5 (44.7-52.2) | 1,200  47.6 (43.6-51.7) | 1,201  50.9 (47.4-54.5) | 1,090  51.4 (47.3-55.5) |
| Education  (aged 30-59 years) | ≤High school | 1,509  44.4 (41.0-47.8) | 1,462  52.2 (48.4-56.0) | 1,555  55.4 (51.7-59.2) | 1,449  57.6 (53.3-61.8) | 1,484  59.0 (55.5-62.4) | 1,381  55.1 (51.0-59.3) | 1,184  59.1 (55.1-63.0) |
|  | ≥College | 1,159  40.2 (37.0-43.3) | 1,166  45.1 (41.5-48.7) | 1,611  47.8 (44.9-50.8) | 1,610  49.6 (46.5-52.6) | 1,618  51.3 (48.1-54.6) | 1,706  52.9 (49.9-55.9) | 1,477  54.4 (51.2-57.5) |
| Education  (aged ≥60 years) | ≤Middle school | 1,208  62.1 (58.9-65.4) | 1,237  63.2 (59.5-67.0) | 1,380  66.5 (63.4-69.6) | 1,398  72.5 (69.3-75.6) | 1,400  71.7 (68.4-75.0) | 1,369  69.4 (65.9-73.0) | 1,209  71.8 (68.3-75.3) |
|  | ≥ High school | 498  44.1 (38.9-49.3) | 546  55.2 (50.6-59.8) | 571  51.4 (46.2-56.7) | 635  57.9 (53.1-62.7) | 701  60.0 (54.6-65.4) | 733  55.8 (51.5-60.0) | 745  52.9 (48.6-57.3) |
| Occupation | Non-manual | 837  42.2 (38.2-46.2) | 827  47.0 (42.7-51.3) | 1,071  50.6 (47.4-53.8) | 1,151  51.5 (48.3-54.8) | 1,143  52.8 (49.3-56.4) | 1,163  53.3 (49.6-57.0) | 1,006  54.7 (50.9-58.6) |
|  | Manual | 1,068  44.1 (40.1-48.1) | 1,061  51.4 (47.4-55.5) | 1,205  55.3 (51.4-59.2) | 1,130  54.4 (49.9-59.0) | 1,240  55.2 (51.4-58.9) | 1,125  54.0 (49.5-58.4) | 964  56.7 (52.7-60.7) |
|  | Others | 763  40.7 (36.5-44.8) | 737  47.5 (43.1-51.9) | 891  48.7 (44.7-52.6) | 780  52.6 (48.4-56.9) | 715  52.5 (47.9-57.1) | 794  54.8 (50.4-59.2) | 688  57.3 (53.1-61.4) |
