# Supplementary material for: Smoking, drinking, and physical activity among Korean adults before and during the COVID-19 pandemic: a special report of the 2020 Korea National Health and Nutrition Examination Survey
Source: Epidemiol Health. 2022 Apr 25;44:e2022043. doi: 10.4178/epih.e2022043 (PMC9133597; doi:10.4178/epih.e2022043)
Supplement: Supplementary Material 8 — Numbers and age-standardized rates (%) of inadequate physical activity by demographic and socioeconomic indicators among Korean men aged 19 or older in the 2014-2020 Korea National Health and Nutrition Examination Survey. [file epih-44-e2022043-suppl8.docx]

Supplementary Material 8. Numbers and age-standardized rates (%) of inadequate physical activity by demographic and socioeconomic indicators among Korean men aged 19 or older in the 2014-2020 Korea National Health and Nutrition Examination Survey.

|  |  | 2014 | 2015 | 2016 | 2017 | 2018 | 2019 | 2020 |
| --- | --- | --- | --- | --- | --- | --- | --- | --- |
| Total |  | 2,069  38.0 (35.5-40.4) | 2,176  44.2 (41.5-47.0) | 2,483  47.5 (44.8-50.2) | 2,568  49.4 (46.8-52.1) | 2,600  49.0 (46.4-51.5) | 2,616  47.4 (44.9-49.9) | 2,433  51.7 (49.1-54.2) |
| Age | 19-29 | 233  20.2 (14.8-25.7) | 312  27.9 (21.8-33.9) | 289  33.1 (26.2-40.1) | 336  33.0 (27.3-38.6) | 353  30.3 (23.8-36.9) | 375  31.6 (26.1-37.0) | 389  38.2 (32.4-44.1) |
|  | 30-39 | 358  41.8 (36.5-47.1) | 270  45.7 (38.3-53.1) | 436  49.3 (44.3-54.3) | 381  48.7 (42.3-55.1) | 405  44.7 (38.3-51.1) | 405  41.5 (35.7-47.3) | 327  51.0 (45.1-57.0) |
|  | 40-49 | 346  42.5 (37.2-47.7) | 365  44.9 (39.1-50.7) | 479  49.3 (43.9-54.7) | 464  51.6 (46.3-57.0) | 456  51.2 (46.5-55.9) | 463  52.2 (47.4-57.1) | 393  56.0 (51.5-60.6) |
|  | 50-59 | 387  39.8 (34.4-45.3) | 443  51.7 (45.9-57.4) | 426  55.3 (49.9-60.7) | 509  54.1 (49.1-59.1) | 469  63.5 (58.2-68.8) | 458  59.1 (54.2-64.0) | 458  59.7 (53.8-65.6) |
|  | 60-69 | 391  46.3 (40.5-52.2) | 435  53.4 (47.9-58.9) | 426  49.1 (44.2-53.9) | 446  65.6 (60.3-70.9) | 480  59.2 (53.9-64.4) | 468  55.6 (50.7-60.5) | 449  55.6 (50.6-60.7) |
|  | 70+ | 354  51.8 (46.2-57.4) | 351  61.7 (55.5-67.8) | 427  64.4 (59.2-69.5) | 432  65.2 (59.8-70.6) | 437  72.3 (66.7-77.9) | 447  67.1 (62.2-72.0) | 417  61.2 (55.4-66.9) |
| Number of household members | 1 | 155  - - | 195  - - | 242  44.0 (36.3-51.6) | 314  49.9 (43.3-56.5) | 297  46.7 (39.2-54.2) | 307  49.9 (41.7-58.1) | 312  50.9 (43.6-58.3) |
|  | 2+ | 1,914  38.1 (35.6-40.7) | 1,981  44.9 (42.0-47.8) | 2,241  47.9 (45.1-50.8) | 2,254  49.3 (46.5-52.1) | 2,303  49.3 (46.7-51.9) | 2,309  47.2 (44.5-49.9) | 2,121  51.8 (48.9-54.6) |
| Residential area | Urban areas | 1,658  36.1 (33.5-38.7) | 1,742  42.4 (39.4-45.5) | 2,004  45.9 (43.0-48.9) | 2,092  48.6 (45.8-51.5) | 2,124  48.3 (45.5-51.2) | 2,086  46.1 (43.4-48.8) | 1,927  50.5 (47.6-53.3) |
|  | Rural areas | 411  45.0 (38.5-51.6) | 434  51.0 (42.9-59.1) | 479  56.2 (49.6-62.9) | 476  53.7 (45.7-61.6) | 476  50.4 (43.6-57.3) | 530  54.5 (46.8-62.2) | 506  58.3 (50.9-65.7) |
| Income | Lowest | 400  40.1 (34.7-45.5) | 422  44.4 (38.7-50.1) | 502  56.9 (51.6-62.2) | 507  50.3 (45.3-55.2) | 517  51.6 (47.0-56.2) | 513  51.4 (45.8-57.0) | 466  55.7 (49.8-61.7) |
|  | Lower middle | 419  39.6 (34.3-44.9) | 431  43.5 (37.2-49.7) | 497  47.3 (42.6-52.0) | 499  51.1 (45.2-57.0) | 513  51.4 (46.3-56.5) | 518  48.8 (43.2-54.5) | 483  53.0 (47.5-58.4) |
|  | Middle | 417  37.3 (31.8-42.8) | 426  47.5 (40.8-54.2) | 481  48.3 (42.9-53.8) | 516  48.9 (44.1-53.7) | 520  53.4 (47.7-59.1) | 509  49.2 (44.2-54.1) | 497  52.1 (46.9-57.3) |
|  | Upper middle | 417  34.6 (29.6-39.6) | 440  47.3 (41.2-53.3) | 494  45.5 (40.2-50.8) | 515  48.8 (43.2-54.4) | 520  45.4 (40.2-50.6) | 533  41.9 (37.2-46.5) | 492  51.8 (45.8-57.9) |
|  | Highest | 413  38.5 (33.3-43.6) | 447  38.5 (32.8-44.2) | 503  39.6 (33.9-45.4) | 527  48.1 (42.7-53.5) | 524  42.4 (37.9-46.8) | 533  45.7 (40.9-50.5) | 489  46.1 (39.9-52.3) |
| Education  (aged 30-59 years) | ≤High school | 541  43.4 (38.3-48.5) | 545  52.6 (46.6-58.6) | 564  57.1 (51.5-62.7) | 582  58.1 (52.4-63.8) | 563  58.9 (54.0-63.7) | 533  53.9 (47.9-60.0) | 486  59.1 (54.2-64.1) |
|  | ≥College | 549  40.1 (35.7-44.6) | 533  41.6 (36.6-46.5) | 776  45.2 (41.2-49.1) | 767  46.1 (41.7-50.5) | 767  47.6 (43.7-51.6) | 792  46.7 (42.8-50.6) | 692  52.3 (47.8-56.9) |
| Education  (aged ≥60 years) | ≤Middle school | 419  57.6 (52.0-63.2) | 428  60.2 (53.7-66.8) | 481  63.1 (58.4-67.8) | 477  73.3 (68.7-77.8) | 472  71.9 (66.7-77.0) | 475  68.6 (63.5-73.7) | 416  72.4 (67.5-77.2) |
|  | ≥ High school | 317  37.6 (31.6-43.7) | 357  53.1 (47.6-58.6) | 368  47.3 (41.2-53.3) | 397  56.6 (50.2-63.0) | 440  57.5 (51.3-63.8) | 438  52.6 (47.2-58.0) | 448  46.9 (41.8-52.1) |
| Occupation | Non-manual | 458  40.1 (35.2-45.1) | 428  43.2 (37.6-48.9) | 579  47.4 (42.9-52.0) | 600  48.9 (44.5-53.4) | 584  48.4 (43.7-53.1) | 588  48.1 (43.8-52.4) | 507  52.4 (46.8-58.0) |
|  | Manual | 552  41.5 (36.7-46.4) | 539  50.9 (45.8-56.0) | 638  54.5 (50.4-58.7) | 626  53.2 (47.7-58.8) | 638  53.5 (48.8-58.2) | 609  52.8 (47.4-58.2) | 540  56.7 (51.9-61.4) |
|  | Others | 79  - - | 109  - - | 124  42.0 (31.6-52.4) | 124  47.5 (36.8-58.1) | 104  54.5 (44.8-64.1) | 124  47.4 (37.3-57.5) | 130  57.3 (47.5-67.1) |
