# Supplementary material for: Smoking, drinking, and physical activity among Korean adults before and during the COVID-19 pandemic: a special report of the 2020 Korea National Health and Nutrition Examination Survey
Source: Epidemiol Health. 2022 Apr 25;44:e2022043. doi: 10.4178/epih.e2022043 (PMC9133597; doi:10.4178/epih.e2022043)
Supplement: Supplementary Material 9 — Numbers and age-standardized rates (%) of inadequate physical activity by demographic and socioeconomic indicators among Korean women aged 19 or older in the 2014-2020 Korea National Health and Nutrition Examination Survey. [file epih-44-e2022043-suppl9.docx]

Supplementary Material 9. Numbers and age-standardized rates (%) of inadequate physical activity by demographic and socioeconomic indicators among Korean women aged 19 or older in the 2014-2020 Korea National Health and Nutrition Examination Survey.

|  |  | 2014 | 2015 | 2016 | 2017 | 2018 | 2019 | 2020 |
| --- | --- | --- | --- | --- | --- | --- | --- | --- |
| Total |  | 2,904  45.3 (42.8-47.8) | 2,889  50.2 (47.7-52.8) | 3,318  53.6 (51.2-56.0) | 3,242  53.4 (51.1-55.7) | 3,362  56.0 (53.6-58.4) | 3,302  57.3 (54.8-59.8) | 2,965  57.0 (54.7-59.4) |
| Age | 19-29 | 348  35.9 (29.7-42.1) | 333  39.2 (33.7-44.7) | 378  43.6 (37.7-49.4) | 368  36.1 (30.2-42.0) | 395  42.9 (36.9-48.8) | 348  44.9 (38.6-51.3) | 389  46.5 (40.7-52.3) |
|  | 30-39 | 504  42.2 (37.0-47.4) | 425  51.7 (45.9-57.5) | 600  52.6 (48.3-56.8) | 476  56.4 (51.8-61.1) | 476  54.2 (48.7-59.7) | 477  61.0 (55.8-66.3) | 406  54.9 (49.2-60.7) |
|  | 40-49 | 504  43.1 (38.4-47.9) | 519  46.0 (41.7-50.3) | 602  55.0 (50.5-59.5) | 597  53.4 (48.8-57.9) | 625  57.2 (52.6-61.8) | 613  57.1 (52.7-61.5) | 523  58.3 (53.8-62.8) |
|  | 50-59 | 572  47.4 (42.6-52.1) | 608  55.8 (51.2-60.3) | 626  54.0 (49.6-58.3) | 639  57.0 (52.8-61.2) | 671  60.8 (56.4-65.2) | 673  58.1 (53.6-62.5) | 554  60.5 (55.8-65.2) |
|  | 60-69 | 500  57.3 (51.8-62.7) | 504  55.1 (49.7-60.5) | 545  59.0 (53.9-64.1) | 592  62.9 (58.0-67.8) | 585  63.4 (58.4-68.3) | 603  65.5 (60.8-70.1) | 599  63.2 (58.4-68.0) |
|  | 70+ | 476  70.9 (66.6-75.3) | 500  75.8 (71.3-80.4) | 567  76.1 (71.8-80.3) | 570  78.0 (73.9-82.2) | 610  79.5 (75.6-83.4) | 588  71.4 (66.9-75.9) | 494  77.7 (73.6-81.8) |
| Number of household members | 1 | 331  - - | 348  - - | 408  57.5 (49.2-65.8) | 444  - - | 475  52.4 (44.6-60.1) | 471  - - | 393  50.8 (41.9-59.8) |
|  | 2+ | 2,573  45.5 (43.0-48.0) | 2,541  50.2 (47.6-52.8) | 2,910  53.4 (51.0-55.8) | 2,798  53.5 (51.1-55.9) | 2,887  56.3 (53.8-58.8) | 2,831  57.3 (54.6-59.9) | 2,572  57.4 (54.9-59.9) |
| Residential area | Urban areas | 2,385  43.4 (40.9-46.0) | 2,375  48.5 (45.8-51.2) | 2,681  52.4 (49.9-54.9) | 2,671  52.1 (49.7-54.4) | 2,778  54.2 (51.6-56.7) | 2,668  55.5 (53.0-58.1) | 2,393  55.0 (52.6-57.5) |
|  | Rural areas | 519  57.1 (47.8-66.4) | 514  60.5 (52.1-68.9) | 637  61.3 (52.9-69.7) | 571  60.9 (53.8-68.1) | 584  68.7 (62.9-74.4) | 634  66.4 (57.8-75.1) | 572  70.3 (64.5-76.2) |
| Income | Lowest | 562  48.1 (43.0-53.3) | 551  51.3 (45.0-57.5) | 643  57.5 (52.6-62.3) | 631  54.9 (50.2-59.6) | 657  59.2 (54.6-63.9) | 641  59.6 (54.4-64.7) | 562  61.2 (55.9-66.5) |
|  | Lower middle | 577  43.1 (38.5-47.6) | 580  51.7 (46.6-56.8) | 664  58.4 (53.8-63.1) | 651  54.4 (49.4-59.4) | 677  56.7 (52.1-61.3) | 651  58.3 (52.9-63.6) | 591  56.8 (51.6-62.0) |
|  | Middle | 583  47.9 (42.9-52.9) | 587  50.0 (45.2-54.8) | 678  54.2 (49.7-58.7) | 648  55.3 (50.1-60.6) | 674  55.8 (50.6-61.1) | 662  56.7 (52.0-61.5) | 602  56.1 (50.6-61.5) |
|  | Upper middle | 590  44.7 (39.8-49.5) | 589  52.4 (47.4-57.3) | 662  47.2 (42.1-52.3) | 653  53.8 (48.8-58.9) | 668  54.6 (49.5-59.7) | 667  56.7 (51.9-61.5) | 599  54.5 (49.3-59.7) |
|  | Highest | 579  43.1 (37.8-48.3) | 570  46.3 (41.0-51.5) | 663  50.6 (45.9-55.2) | 647  48.7 (44.0-53.4) | 676  53.3 (48.0-58.6) | 668  56.2 (51.5-61.0) | 601  56.7 (51.9-61.4) |
| Education  (aged 30-59 years) | ≤High school | 968  45.1 (40.6-49.6) | 917  52.0 (46.9-57.0) | 991  54.0 (49.6-58.3) | 867  57.1 (51.8-62.4) | 921  59.3 (54.4-64.1) | 848  57.0 (52.0-62.1) | 698  58.5 (52.9-64.1) |
|  | ≥College | 610  39.9 (35.6-44.2) | 633  49.7 (44.9-54.4) | 835  51.5 (47.8-55.1) | 843  53.7 (49.9-57.5) | 851  55.6 (51.4-59.8) | 914  60.0 (56.1-63.8) | 785  56.6 (52.6-60.5) |
| Education  (aged ≥60 years) | ≤Middle school | 789  64.4 (60.1-68.7) | 809  64.5 (60.0-69.0) | 899  68.2 (64.1-72.2) | 921  71.6 (67.6-75.5) | 928  71.4 (67.6-75.2) | 894  69.9 (65.9-74.0) | 793  71.2 (66.6-75.8) |
|  | ≥ High school | 181  58.3 (50.3-66.2) | 189  60.0 (51.1-68.8) | 203  59.3 (50.3-68.3) | 238  60.6 (53.6-67.6) | 261  64.2 (56.7-71.7) | 295  60.3 (54.1-66.6) | 297  63.7 (56.0-71.4) |
| Occupation | Non-manual | 379  46.9 (40.4-53.5) | 399  52.1 (46.2-58.0) | 492  56.0 (51.4-60.6) | 551  55.5 (50.9-60.1) | 559  59.3 (54.7-63.9) | 575  60.2 (55.6-64.8) | 499  57.9 (52.9-63.0) |
|  | Manual | 516  48.2 (42.2-54.3) | 522  52.2 (45.7-58.7) | 567  56.4 (49.8-63.0) | 504  57.9 (50.9-65.0) | 602  59.0 (52.3-65.7) | 516  56.8 (50.5-63.0) | 424  55.8 (49.2-62.4) |
|  | Others | 684  39.6 (35.4-43.9) | 628  48.0 (43.3-52.6) | 767  49.6 (45.7-53.6) | 656  53.7 (49.1-58.2) | 611  52.4 (47.3-57.4) | 670  57.8 (53.6-62.1) | 558  57.3 (52.8-61.9) |
