# Supplementary material for: Smoking, drinking, and physical activity among Korean adults before and during the COVID-19 pandemic: a special report of the 2020 Korea National Health and Nutrition Examination Survey
Source: Epidemiol Health. 2022 Apr 25;44:e2022043. doi: 10.4178/epih.e2022043 (PMC9133597; doi:10.4178/epih.e2022043)
Supplement: Supplementary Material 10 — Differences between periods and annual percentage changes of current cigarette smoking by demographic and socioeconomic indicators among Koreans (men and women combined) aged 19 or older in the 2011-2020 Korea National Health and Nutrition Examination Survey. [file epih-44-e2022043-suppl10.docx]

Supplementary Material 10. Differences between periods and annual percentage changes of current cigarette smoking by demographic and socioeconomic indicators among Koreans (men and women combined) aged 19 or older in the 2011-2020 Korea National Health and Nutrition Examination Survey.

|  |  | Diff. btw.  2011-2019 and 2020 | Diff. btw.  2017-2019 and 2020 | Diff. btw.  2019 and  2020 | Annual percentage change |
| --- | --- | --- | --- | --- | --- |
| Total |  | -3.2 (-4.9- -1.4)* | -1.5 (-3.5- 0.4) | -0.9 (-3.3- 1.5) | -2.6 (-3.3~ -1.9)* |
| Age | 19-29 | -3.1 (-6.6- 0.4) | -2.4 (-6.4- 1.5) | -2.9 (-8.0- 2.2) | -1.9 (-3.4- -0.4)* |
|  | 30-39 | -6.2 (-10.1- -2.3)* | -1.8 (-6.0- 2.5) | -1.1 (-6.3- 4.1) | -4.7 (-5.9- -3.6)* |
|  | 40-49 | -2.1 (-5.5- 1.3) | -1.2 (-4.9- 2.6) | 1.6 (-2.8- 6.1) | -1.4 (-3.0- 0.3) |
|  | 50-59 | -2.8 (-5.6- 0.1) | -1.9 (-5.1- 1.3) | -1.1 (-5.0- 2.8) | -2.0 (-3.5- -0.5)* |
|  | 60-69 | -0.1 (-3.0- 2.8) | 0.2 (-3- 3.3) | -1.3 (-5.4- 2.8) | -0.9 (-3.7- 2.0) |
|  | 70+ | -2.1 (-4.1- -0.1)* | -0.6 (-2.8- 1.5) | -1.2 (-3.8- 1.4) | -5.6 (-8.9- -2.2)* |
| Number of household members | 1 | -0.4 (-6.3- 5.6) | -0.4 (-6.8- 6.1) | -0.8 (-8.5- 6.9) | -1.0 (-3.2- 1.1) |
|  | 2+ | -3.8 (-5.5- -2.1)* | -1.7 (-3.6- 0.2) | -1.0 (-3.4- 1.4) | -3.2 (-3.9- -2.5)* |
| Residential area | Urban areas | -2.4 (-4.3- -0.5)* | -0.8 (-2.9- 1.3) | -0.6 (-3.3- 2.1) | -2.4 (-3.1- -1.8)* |
|  | Rural areas | -8.3 (-12.4- -4.1)* | -6.2 (-10.9- -1.6)* | -2.3 (-7.4- 2.9) | -3.4 (-5.9- -0.8)* |
| Income | Lowest | -4.1 (-7.9- -0.4)* | -2.6 (-6.7- 1.4) | -4.2 (-9.0- 0.6) | -2.6 (-4.0- -1.1)* |
|  | Lower middle | -1.9 (-5.3- 1.5) | -1.0 (-4.8- 2.8) | 1.8 (-2.9- 6.4) | -2.1 (-4.0- -0.2)* |
|  | Middle | -4.2 (-7.8- -0.5)* | -3.1 (-7.1- 0.9) | -4.2 (-9.1- 0.8) | -2.0 (-3.4- -0.6)* |
|  | Upper middle | -3.0 (-6.4- 0.4) | -2.1 (-5.8- 1.6) | -1.6 (-6.2- 3.0) | -1.4 (-2.7- -0.1)* |
|  | Highest | -2.1 (-5.7- 1.5) | 1.7 (-2.1- 5.5) | 3.4 (-0.9- 7.8) | -4.7 (-7.0- -2.4)* |
| Education  (aged 30-59 years) | ≤High school | 0.0 (-4.0- 4.1) | -1.3 (-5.7- 3.1) | 0.0 (-5.3- 5.4) | 0.2 (-0.9- 1.3) |
|  | ≥College | -5.6 (-8.0- -3.2)* | -2.2 (-4.9- 0.5) | -1.1 (-4.4- 2.2) | -5.2 (-6.9- -3.4)* |
| Education  (aged ≥60 years) | ≤Middle school | -0.6 (-3.2- 2.1) | 0.1 (-2.7- 3.0) | -0.7 (-4.2- 2.8) | -3.0 (-5.9- -0.1)* |
|  | ≥ High school | -2.5 (-5.5- 0.5) | -1.1 (-4.4- 2.2) | -2.2 (-6.3- 2.0) | -3.3 (-6.3- -0.1)* |
| Occupation | Non-manual | -6.1 (-9.2- -2.9)* | -1.7 (-5.1- 1.7) | -0.2 (-4.4- 4.0) | -5.9 (-8.4- -3.3)* |
|  | Manual | -4.1 (-8.4- 0.2) | -3.2 (-7.9- 1.5) | -0.1 (-5.8- 5.6) | -1.5 (-2.6- -0.4)* |
|  | Others | 0.5 (-2.8- 3.8) | 0.7 (-2.9- 4.3) | -0.3 (-4.8- 4.1) | 0.3 (-2.8- 3.4) |

*p<0.05
