# Supplementary material for: Smoking, drinking, and physical activity among Korean adults before and during the COVID-19 pandemic: a special report of the 2020 Korea National Health and Nutrition Examination Survey
Source: Epidemiol Health. 2022 Apr 25;44:e2022043. doi: 10.4178/epih.e2022043 (PMC9133597; doi:10.4178/epih.e2022043)
Supplement: Supplementary Material 11 — Differences between periods and annual percentage changes of high-risk drinking by demographic and socioeconomic indicators among Koreans (men and women combined) aged 19 or older in the 2011-2020 Korea National Health and Nutrition Examination Survey. [file epih-44-e2022043-suppl11.docx]

Supplementary Material 11. Differences between periods and annual percentage changes of high-risk drinking by demographic and socioeconomic indicators among Koreans (men and women combined) aged 19 or older in the 2011-2020 Korea National Health and Nutrition Examination Survey.

|  |  | Diff. btw.  2011-2019 and  2020 | Diff. btw.  2017-2019 and 2020 | Diff. btw.  2019 and  2020 | Annual percentage change |
| --- | --- | --- | --- | --- | --- |
| Total |  | 0.4 (-1.0- 1.7) | 0.2 (-1.2- 1.7) | 1.5 (-0.3- 3.3) | 0.2 (-1.1~ 1.6) |
| Age | 19-29 | -2.2 (-5.1- 0.7) | -2.3 (-5.6- 0.9) | 0.3 (-3.6- 4.1) | -1.3 (-4.1- 1.7) |
|  | 30-39 | 0.2 (-3.0- 3.3) | 1.2 (-2.2- 4.6) | 2.7 (-1.5- 7.0) | -1.7 (-3.3- 0.0)* |
|  | 40-49 | 2.6 (-0.7- 5.9) | 2.2 (-1.4- 5.7) | 3.6 (-0.4- 7.7) | 1.0 (-0.8- 2.9) |
|  | 50-59 | -0.2 (-2.7- 2.3) | -0.5 (-3.1- 2.2) | 0.0 (-3.3- 3.2) | 0.5 (-1.1- 2.2) |
|  | 60-69 | 2.5 (0.4- 4.5)* | 0.7 (-1.6- 3.0) | 0.5 (-2.2- 3.3) | 7.3 (3.9- 10.9)* |
|  | 70+ | 0.5 (-0.7- 1.7) | -0.2 (-1.6- 1.1) | -1.3 (-3.1- 0.5) | 7.1 (1.4- 13.2)* |
| Number of household members | 1 | -0.2 (-4.8- 4.3) | 0.6 (-4.3- 5.5) | -2.2 (-9.1- 4.7) | -2.1 (-5.3- 1.1) |
|  | 2+ | 0.3 (-1.1- 1.8) | 0.2 (-1.4- 1.8) | 1.7 (-0.1- 3.6) | 0.2 (-1.4- 1.7) |
| Residential area | Urban areas | 0.5 (-1.0- 2.0) | 0.3 (-1.3- 1.9) | 1.8 (-0.2- 3.8) | 0.2 (-1.2- 1.7) |
|  | Rural areas | -0.4 (-3.8- 3.1) | -0.2 (-4.0- 3.6) | -0.6 (-4.9- 3.6) | 0.3 (-2.1- 2.9) |
| Income | Lowest | -0.9 (-3.5- 1.8) | -1 (-3.9- 1.8) | -1 (-4.5- 2.5) | -0.3 (-2.5- 1.9) |
|  | Lower middle | 0.4 (-2.2- 3.1) | -0.8 (-3.7- 2.1) | -0.2 (-4.0- 3.6) | 1.7 (-0.3- 3.8) |
|  | Middle | 1.7 (-1.4- 4.8) | 1.1 (-2.3- 4.5) | 2.7 (-1.3- 6.6) | 0.1 (-3.4- 3.7) |
|  | Upper middle | -0.6 (-3.6- 2.4) | -0.4 (-3.7- 2.9) | 2.2 (-1.4- 5.8) | 0.2 (-3.0- 3.6) |
|  | Highest | 1.5 (-1.3- 4.3) | 2.5 (-0.5- 5.6) | 3.6 (-0.2- 7.3) | -1.1 (-3.5- 1.5) |
| Education  (aged 30-59 years) | ≤High school | 3.4 (0.1- 6.8)* | 1.7 (-2.0- 5.3) | 4.2 (-0.2- 8.5) | 2.3 (-0.1- 4.7) |
|  | ≥College | -0.3 (-2.6- 2.0) | 0.7 (-1.8- 3.1) | 1.2 (-1.8- 4.2) | -1.9 (-3.8- -0.1)* |
| Education  (aged ≥60 years) | ≤Middle school | -0.2 (-1.8- 1.3) | -1.7 (-3.5- 0.1) | -1.6 (-3.9- 0.6) | 5.3 (0.2- 10.7)* |
|  | ≥ High school | 1.8 (-0.5- 4.1) | 0.6 (-2.0- 3.1) | -0.6 (-3.7- 2.6) | 8.1 (4.2- 12.1)* |
| Occupation | Non-manual | -1.6 (-4.4- 1.3) | 0.2 (-2.8- 3.2) | 1.6 (-2.2- 5.4) | -3.1 (-5.0- -1.2)* |
|  | Manual | 2.2 (-1.7- 6.2) | 1.7 (-2.5- 5.9) | 2.5 (-2.5- 7.5) | 0.6 (-1.4- 2.7) |
|  | Others | 2.8 (-0.2- 5.8) | 2.1 (-1.1- 5.3) | 3.6 (-0.1- 7.3) | 4.3 (-1.0- 9.9) |

*p<0.05
